# Inequity in uptake of maternal health care services in developing countries: a systematic review and meta-analysis

**DOI:** 10.3389/fpubh.2024.1415092

**Published:** 2024-06-26

**Authors:** Addisu Alemayehu Gube, Edit Murányi, Jozsef Vitrai, Szimonetta Lohner

**Affiliations:** ^1^Doctoral School of Health Sciences, Faculty of Health Sciences, University of Pécs, Pécs, Hungary; ^2^Department of Public Health Medicine, Medical School, University of Pécs, Pécs, Hungary; ^3^Cochrane Hungary, Medical School, University of Pécs, Pécs, Hungary

**Keywords:** inequity, maternal health care services, developing countries, systematic review, meta-analysis, low-or middle-income countries

## Abstract

**Background:**

Maternal health service uptake remains an important predictor of maternal outcomes including maternal mortality. This systematic review and meta-analysis aimed to summarize the available evidence on the uptake of maternal health care services in developing countries and to assess the impact of place of residence, education status, and wealth index on the uptake of these services.

**Methods:**

We examined the databases MEDLINE, Web of Science, Global Index Medicus, and Scopus until June 14, 2022. Cross-sectional studies done between 2015 and 2022 were considered. Mothers of reproductive age and all states of health were included in the study. Independently, two authors determined the eligibility of studies, extracted data, evaluated the risk of bias, and ranked the evidence’s degree of certainty. To combine the data, we performed a random-effects meta-analysis. The PROSPERO registration ID is CRD42022304094.

**Results:**

We included 51 studies. Mothers living in urban areas were three times more likely to receive antenatal care (OR 2.95; 95% CI 2.23 to 3.89; 15 studies; 340,390 participants) than rural mothers. Compared with no education, those with primary education were twice as likely to utilize antenatal care (OR 2.36; 95% CI 1.80 to 3.09; 9 studies; 154,398 participants) and those with secondary and higher education were six and fourteen times more likely to utilize antenatal care, respectively. Mothers in the second wealth index were twice as likely as mothers in the lowest wealth index to utilize antenatal care (OR 1.62; 95% CI 1.36 to 1.91; 10 studies; 224,530 participants) and antenatal care utilization increased further among mothers in the higher wealth index. We observed similar relative inequalities in skilled delivery care and postnatal care utilization based on the pace of residence, education, and wealth index.

**Conclusion:**

In developing countries, the problem of inequity in utilizing maternal health care services persists and needs considerable attention.

## Introduction

Health inequality refers to a measurable aspect of difference in health that can be observed across individuals or social groups (1). These differences might exist in health conditions or might be related to access to health prevention, therapy, or rehabilitation (2). The term ‘health inequity’ involves the moral social perception of existing health inequalities and refers to inequalities that are considered unjust by society or that stem from some kind of injustice, mainly affecting groups with less wealth, prestige, and power (3, 4).

Maternal mortality, defined as death during pregnancy or within 42 days after childbirth, is an important indicator of socioeconomic inequalities, as it is influenced by the availability of health care and obstetric care (5). Antenatal care contacts and births attended by skilled health professionals, early routine postnatal care, and timely management and treatment of complications are important aspects of reducing preventable maternal mortality (6, 7).

As part of the Sustainable Development Goals (SDGs), the member states of the United Nations expressed their commitment to reducing maternal mortality to less than 70 maternal deaths per 100,000 live births by 2030 (8). Based on recent data, we are far from reaching these goals, as since 2016 maternal mortality has been reported to decrease in only a part of the world, including Central and South Asia, Australia, and New Zealand, while in Sub-Saharan Africa, Oceania, East and Southeast Asia, and north Africa stagnation in maternal mortality is observed (9). In the same period, maternal mortality increased in Europe, North America, Latin America, and the Caribbean (9). Infectious diseases, adolescent pregnancies, cesarean section, availability of health workforce, coverage of births by health facilities and hospitals, and inequalities in service coverage might play the most important role in maternal mortality (10). Underutilization of maternal and child health care services can put women and their offspring at risk of dying (11).

Worldwide, governments have started to place increasing emphasis on addressing inequities in maternal and child health services and these efforts improved relative equity of the coverage of reproductive and maternal health services across countries over the last decades (12). However, among low and middle-income countries there are still differences in the extent to which equity is reached, and inequity in coverage of health services persists in some of the countries more than in others (13). For the current 2024 fiscal year, low-income economies are defined as those with a Gross National Income (GNI) *per capita*, calculated using the World Bank Atlas method, of $1,135 or less in 2022; lower middle-income economies are those with a GNI *per capita* between $1,136 and $4,465; upper middle-income economies are those with a GNI *per capita* between $4,466 and $13,845; high-income economies are those with a GNI *per capita* of $13,846 or more. And low-or middle-income countries are referred to as developing countries (14, 15).

Even across different regions of a country, significant differences might be present in the utilization of maternal health services (16). The most important factors indicated in studies to have an important impact on the utilization of maternal health services were education, region of residence, maternal age, and distance to health facilities (17). For example, if a woman lives in an impoverished rural area, like Sub-Saharan Africa and South Asia, where the number of available skilled health professionals is limited, the probability of not receiving sufficient healthcare is extremely high. In high- and upper-middle-income countries more than 90% of all births are attended by a trained midwife, doctor, or nurse. In contrast, in low-income and lower-middle-income countries less than half of all births are assisted by such skilled health personnel (18). According to World Health Organization (WHO), childbearing or reproductive age for women is from 15 to 49 years (19).

A previous systematic review carried out on equity in maternal health care service utilization in developing countries identified 36 studies published between 2005 and 2015, out of which 33 reported severe inequities in maternal health care utilization (20). This systematic review included only studies published in English, and maternal health care services were limited to antenatal care. Data were summarized narratively but not quantitatively. Besides, new studies have been published since then, which might show a more up-to-date picture.

For the present systematic review and meta-analysis, maternal health care services considered based on the literature (21) are antenatal care, skilled delivery care, and postnatal care, while inequity for the services is assessed based on three variables including residence, wealth index, and educational status.

This systematic review and meta-analysis aimed to summarize available evidence related to the uptake of maternal healthcare services in developing countries in the period of 2015 to 2022 and to assess differences across groups with different places of residence, education status, and wealth index.

## Methods

The methodology and the results are reported according to the Preferred Reporting Items for Systematic Reviews and MetaAnalyses (PRISMA) reporting guideline (22). This study is registered on PROSPERO with the registration ID: CRD42022304094.

### Searches

For this systematic review and meta-analysis, we searched the following electronic databases until 14th June 2022 without restrictions to the language of publications: Ovid MEDLINE (09/06/2022), Web of Science (comprising Science Citation Index and Emerging Citation Index) (14/06/2022), Global Index Medicus comprising African Index Medicus (AIM), Index Medicus for the Eastern Mediterranean Region (IMEMR), Index Medicus for the South-east Asia Region (IMSEAR), Latin America and the Caribbean Literature on Health Science (LILACS) and Western Pacific Region Index Medicus (WPRO) (16/06/2022) and Scopus (14/06/2022). Details of our search strategies are available in the Supplementary material.

We used a snowball search method to identify other potentially eligible studies or supplementary publications by searching the reference lists of included studies. All 51 studies included were published in English.

### Study inclusion and exclusion criteria

We included cross-sectional (observational) studies investigating maternal health care services (including antenatal care, skilled delivery care, and postnatal care) utilization either separately or all services together from 2015 to 2022 in developing countries. The participants of the studies were mothers of reproductive age (15–49 years) (19) with all health statuses, residing in and having utilized maternal health care services in developing (low and middle-income) countries. Health status refers to medical conditions (both physical and mental health), claims experience, receipt of health care, medical history, genetic information, evidence of insurability, and disability (23).

A country’s development status was determined using the World Bank Classifications of countries (14).

Using Covidence^™^ software, two review authors (GA and EM) separately screened the titles and abstracts of each retrieved record. In a subsequent step, all possibly relevant full texts were evaluated for eligibility. Any differences of opinion were settled by consensus or by consulting a third author (SL).

### Potential effect modifiers and reasons for heterogeneity

We assessed methodological heterogeneity through the assessment of risk of bias, and clinical heterogeneity through the assessment of similarities and differences between studies in terms of types of participants and outcomes. We considered the size and direction of the effect and used a standard *χ*^2^ test with a significance level of *α* = 0.1 and the *I*^2^ statistic, which quantifies inconsistency across studies, to assess the effect of heterogeneity on the metaanalysis. In almost all socioeconomic sub-groups we observed high heterogeneity among studies.

We used funnel plots to assess reporting bias and investigate small-study effects when at least ten studies were included in a metaanalysis.

### Study quality assessment

Each included study’s risk of bias was evaluated separately by two review authors (GA and EM), and any discrepancies were settled by consensus. The risk of bias was evaluated with the Joanna Briggs Institute (JBI) critical appraisal checklist for analytical cross-sectional studies (24). Based on the checklist, studies were rated on an 8-point scale (Table 1). Those studies achieving a score exceeding half of the total points were eligible for inclusion in the systematic review and meta-analysis.

**Table 1:** Study quality assessment using the Joanna Briggs Institute critical appraisal checklist for analytical cross-sectional studies.

Studies	(JBI) critical appraisal questions									
	Q1	Q2	Q3	Q4	Q5	Q6	Q7	Q8	Score	Overall appraisal
El-Khatib 2020	Y	Y	Y	Y	N	Y	Y	Y	7	Include
Fagbamigbe 2021	Y	Y	Y	Y	N	U	Y	Y	6	Include
Dankwah 2021	Y	Y	Y	Y	N	N	Y	Y	6	Include
Aziz 2022	Y	Y	Y	Y	Y	Y	Y	Y	8	Include
Ameyaw 2020	Y	Y	Y	N	Y	Y	Y	Y	7	Include
Ahinkorah 2020	Y	Y	Y	Y	Y	Y	Y	Y	8	Include
Bintabara 2021	Y	Y	Y	N	Y	Y	Y	Y	7	Include
Fagbamigbe 2022	Y	Y	Y	N	N	Y	Y	Y	6	Include
Ekholuenetale 2021	Y	Y	Y	N	N	Y	Y	Y	6	Include
Bobo 2017	N	Y	Y	Y	Y	U	Y	Y	6	Include
Daka 2020	Y	Y	Y	N	N	Y	Y	Y	6	Include
Fan 2021	Y	Y	Y	N	Y	N	Y	Y	6	Include
Woldeamanuel 2021	Y	Y	Y	N	Y	U	Y	Y	6	Include
Tesfaye 2017	Y	Y	Y	Y	Y	Y	Y	Y	8	Include
Farraga 2018	Y	Y	Y	N	Y	Y	Y	Y	7	Include
Khatri 2021	Y	Y	Y	N	Y	Y	Y	Y	7	Include
Kien 2019	Y	Y	Y	Y	Y	Y	Y	Y	8	Include
Krishnamoorthy 2020	Y	Y	Y	U	Y	N	Y	Y	6	Include
Mumtaz 2019	Y	Y	Y	N	Y	Y	Y	Y	7	Include
Dinke 2017	Y	Y	Y	Y	Y	Y	Y	Y	8	Include
Kim 2016	Y	Y	N	N	Y	Y	Y	Y	6	Include
Karanja 2018	Y	Y	Y	N	Y	Y	Y	Y	7	Include
Kumar 2019	Y	Y	Y	N	Y	Y	Y	Y	7	Include
Kpodotsi 2021	Y	Y	Y	N	Y	N	Y	Y	6	Include
Ozumba 2019	Y	Y	Y	Y	Y	Y	Y	Y	8	Include
Rahman 2021	Y	N	Y	N	Y	Y	Y	Y	6	Include
Pervin 2021	Y	Y	Y	Y	Y	Y	Y	Y	8	Include
Zegeye 2022	Y	Y	Y	N	Y	U	Y	Y	6	Include
Mwase 2018	Y	Y	N	N	Y	Y	Y	Y	6	Include
Rahman 2017	Y	Y	Y	N	Y	Y	Y	Y	7	Include
Negeri 2021	Y	Y	Y	Y	Y	Y	Y	Y	8	Include
Rosário 2019	Y	Y	N	N	Y	Y	Y	Y	6	Include
Shibre 2020	Y	Y	Y	N	Y	U	Y	Y	6	Include
Yaya 2021	Y	Y	N	Y	Y	Y	Y	Y	7	Include
Lukwa 2022	Y	Y	Y	N	Y	Y	Y	Y	7	Include
Anarwat 2021	Y	Y	Y	Y	N	N	Y	Y	6	Include
Nonyane 2015	Y	Y	Y	Y	Y	N	Y	Y	7	Include
Ghosh 2020	Y	Y	Y	N	Y	Y	Y	Y	7	Include
Gudu 2017	Y	Y	Y	N	Y	U	Y	Y	6	Include
Hodge 2016	Y	Y	N	Y	Y	N	Y	Y	6	Include
Mehata 2017	Y	Y	Y	N	Y	Y	Y	Y	7	Include
Huda 2018	Y	Y	N	Y	Y	N	Y	Y	6	Include
Keats 2018	Y	Y	N	Y	Y	N	Y	Y	6	Include
Laksono 2020	Y	Y	Y	Y	Y	Y	Y	Y	8	Include
Målqvist 2017	Y	Y	Y	Y	Y	U	Y	Y	7	Include
Perera 2021	Y	Y	N	Y	Y	Y	Y	Y	7	Include
Nwosu 2019	Y	Y	N	Y	Y	N	Y	Y	6	Include
Okoli 2020	Y	Y	Y	Y	Y	N	Y	Y	7	Include
Myint 2018	Y	Y	Y	Y	N	U	Y	Y	6	Include
Wang 2021	Y	Y	N	Y	Y	N	Y	Y	6	Include
Dimbuene 2018	Y	Y	Y	Y	Y	N	Y	Y	7	Include

*Y, yes; N, no; U, unclear; Q, question. The score is calculated by summing up Y in each row.

### Data extraction strategy

We retrieved information on study methods, participants, maternal health care services, outcomes, funding sources, and potential conflict of interest statements from full-text publications. One reviewer (GA or EM) extracted the data, while a second reviewer (GA or EM) verified its accuracy, consistency, and completeness.

The outcome of interest for this systematic review was inequity in utilization or uptake of maternal health care services (antenatal care, skilled delivery care, and postnatal care), reported as frequency or percentage by mothers’ residence, educational status, and wealth index.

### Data synthesis and presentation

We conducted a metaanalysis for every outcome for which we judged the participants and outcomes to be similar enough to provide meaningful results. We conducted meta-analyses for the following three comparisons: Antenatal care utilization versus non-utilization, skilled delivery care utilization versus non-utilization, and post-natal care utilization versus non-utilization. To assess inequity, we investigated the following three factors in association with maternal health care utilization: place of residence (urban and rural), educational status (no education, primary, secondary, and higher), and wealth index (lowest, second, middle, fourth and highest).

We presented the results as odds ratios (ORs) with 95% confidence intervals because our data were dichotomous. We did statistical analyses using RevMan (version 5.4.1). As we expected differences between studies like differences in sample size, we decided to combine the data using a randomeffects model. We used Mantel–Haenszel weighting because the outcomes are dichotomous.

Results of eligible studies that do not provide data in an appropriate format for meta-analysis are summarized in a narrative format. The result is presented using PRISMA 2020 checklist (Supplementary material).

## Results

**Figure 1:**
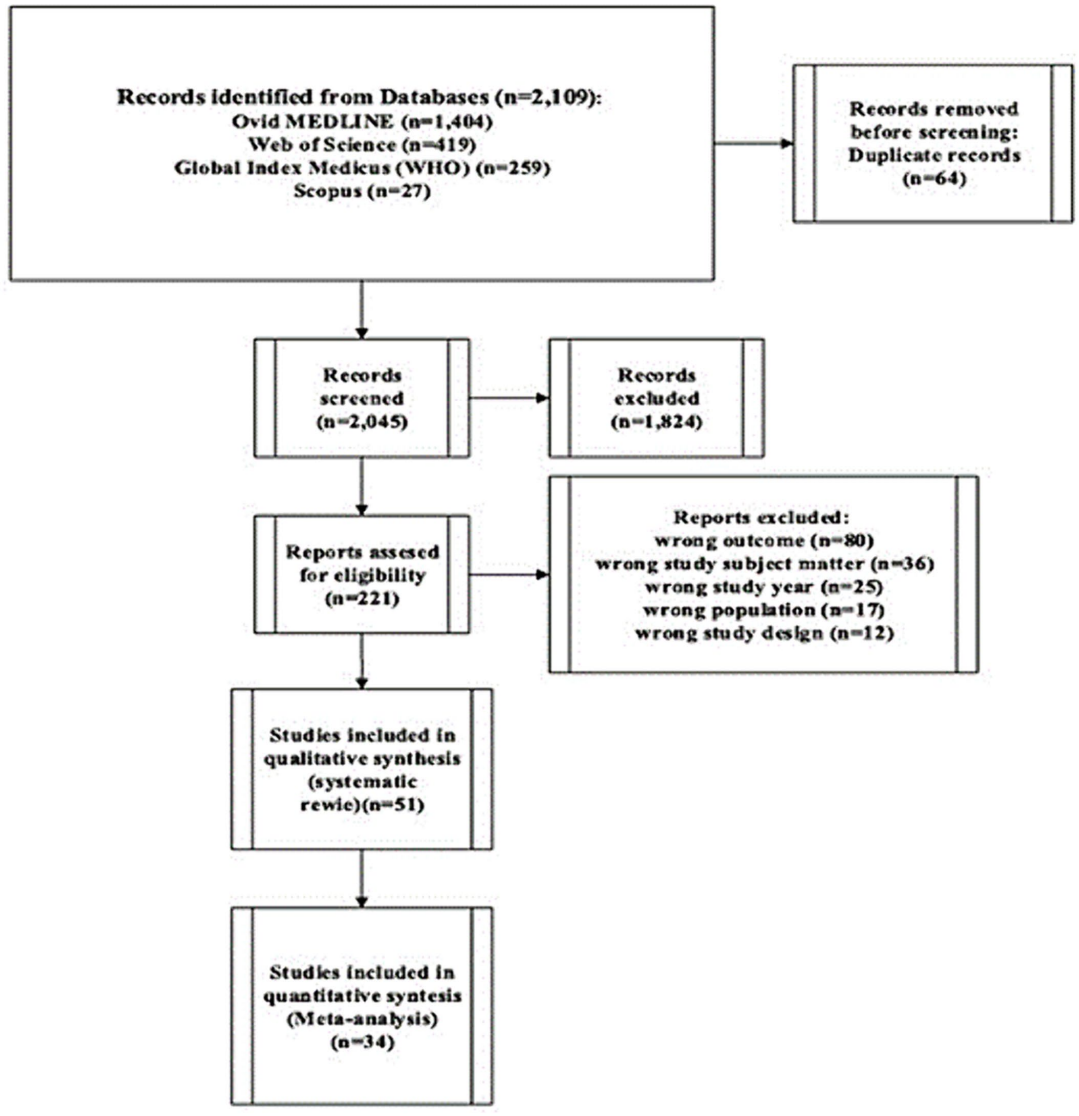
PRISMA flow diagram indicating study selection.

We retrieved 2,109 studies through database searching. We removed duplicates and screened 2,045 studies based on their titles and abstracts. After removing irrelevant studies, we assessed 221 full-text studies for eligibility to be included in this systematic review. Finally, 51 studies met our inclusion criteria. Of these, we were able to include 34 studies in the quantitative syntheses (meta-analysis) (Figure 1).

All studies were conducted in low-income and lower-middle-income countries. The age of the participants in the included studies ranged from 15–49 years. The types of maternal health care services were antenatal care in 14 studies, skilled delivery care in 14 studies, postnatal care in 3 studies, and in another 20 studies, the services were at least two of maternal health care services mentioned above (Table 2).

**Table 2:** Key characteristics of included studies.

Study ID	Reference	Country	Economic level of the country* (14)	Sample size	Age group in years	Type of maternal health care service
El-Khatib 2020	(25)	Nigeria	Lower-middle income	52,654	15–49	Antenatal care
Fagbamigbe 2021	(26)	Nigeria	Lower-middle income	21,785	15–49	Antenatal care
Dankwah 2021	(27)	Ghana	Lower-middle income	4,292	15–49	Postnatal care
Aziz 2022	(28)	Bangladesh	Lower-middle income	4,948	15–49	Postnatal care
Ameyaw 2020	(29)	Mali	Low income	6,502	15–49	Skilled delivery care
Ameyaw 2020	(29)	Niger	Low income	7,432	15–49	Skilled delivery care
Ameyaw 2020	(29)	Sierra Leone	Low income	6,461	15–49	Skilled delivery care
Ahinkorah 2020	(30)	Guinea	Low income	5,046	15–49	Skilled delivery care
Bintabara 2021	(31)	Tanzania	Lower-middle-income	7,079	15–49	Antenatal care
Fagbamigbe 2022	(32)	Nigeria	Lower-middle income	68,679	15–49	Skilled delivery care
Ekholuenetale 2021	(33)	Ghana	Lower-middle income	1,404	15–49	Antenatal care
Bobo 2017 Daka 2020 Fan 2021 Woldeamanuel 2021 Tesfaye 2017	(16, 34–37)	Ethiopia	Low income	3,129	15–49	Antenatal care, Skilled delivery care, and postnatal care
Farraga 2018	(38)	Egypt	Lower-middle income	755	15–49	Antenatal care
Khatri 2021	(39)	Nepal	Lower-middle-income	1978	15–49	Antenatal care, Skilled delivery care, and postnatal care
Kien 2019	(40)	Vietnam	Lower-middle-income	1,475	15–49	Antenatal Care
Krishnamoorthy 2020	(41)	India	Lower-middle-income	190,797	15–49	Antenatal care, Skilled delivery care, and postnatal care
Mumtaz 2019	(42)	Afghanistan	Low income	19,642	15–49	Antenatal care and Skilled delivery care
Dinke 2017	(43)	Ethiopia	Low income	588	15–49	Skilled delivery care
Kim 2016	(44)	Afghanistan	Low income	18,255	15–49	Antenatal care and Skilled delivery care
Karanja 2018	(45)	Kenya	Lower-middle-income	200	15–49	Skilled delivery care
Kumar 2019	(46)	India	Lower-middle-income	190,898	15–49	Antenatal care
Kpodotsi 2021	(47)	Ghana	Lower-middle income	1,305	15–49	Skilled delivery care
Ozumba 2019	(48)	Nigeria	Lower-middle income	1,600	15–49	Antenatal care
Rahman 2021	(49)	Nepal	Lower-middle-income	3,962	15–49	Skilled delivery care
Rahman 2021	(49)	Pakistan	Lower-middle income	8,189	15–49	Skilled delivery care
Rahman 2021	(49)	Bangladesh	Lower-middle income	4,278	15–49	Skilled delivery care
Pervin 2021	(50)	Bangladesh	Lower-middle income	3,293	15–49	Skilled delivery care
Zegeye 2022	(51)	Guinea	Low income	2,779	15–49	Skilled delivery care
Mwase 2018	(52)	Burkina Faso	Low income	6,601 for ANC, 6535 for SDC, and 6,526 for PNC1	15–49	Antenatal care, Skilled delivery care, and postnatal care 1
Rahman 2017	(53)	Bangladesh	Lower-middle income	1,435	15–49	Antenatal care
Negeri 2021	(54)	Ethiopia	Low income	1,200	15–49	Antenatal care
Rosário 2019	(55)	Angola	Lower-middle income	10,084	15–49	Antenatal care
Shibre 2020	(56)	Angola	Lower-middle income	8,492	15–49	Antenatal care
Yaya 2021	(57)	Cameroon	Lower-middle-income	7,881	15–49	Skilled delivery care
Lukwa 2022	(58)	Zimbabwe	Low income		15–49	Antenatal care, Skilled delivery care, and postnatal care
Anarwat 2021	(59)	Ghana	Lower-middle income	10,627	15–49	Prenatal care and skilled delivery care
Nonyane 2015	(60)	Nepal	Lower-middle income	630	15–49	Antenatal and skilled delivery care
Ghosh 2020	(61)	India	Lower-middle income	699,686	15–49	Antenatal and skilled delivery care
Gudu 2017	(62)	Ghana	Lower-middle income	422	15–49	Skilled service delivery
Hodge 2016	(63)	Philippines	Lower-middle income	7,121	15–49	Skilled service delivery
Mehata 2017	(64)	Nepal	Lower-middle income	For ANC (13,211) and institutional delivery (14,969)	15–49	Antenatal care and skilled delivery care
Huda 2018	(65)	Nepal, Pakistan, and Bangladesh	Lower-middle income		15–49	Skilled delivery care
Keats 2018	(66)	Kenya	Lower-middle-income	31, 380	15–49	Antenatal care and skilled delivery care
Laksono 2020	(67)	Indonesia	Lower-middle-income	15,351	15–49	Antenatal care
Målqvist 2017	(68)	Nepal	Lower-middle income	4,745, 4,066, 4,079 and 2086	15–49	Antenatal care and skilled delivery care
Perera 2021	(69)	Sri Lanka	Lower-middle income	8,313	15–49	Postnatal care
Nwosu 2019	(70)	Nigeria	Lower-middle income	18,559	15–49	Antenatal care
Okoli 2020	(71)	Nigeria	Lower-middle income		15–49	Antenatal care and skill delivery care
Myint 2018	(72)	Myanmar	Lower-middle income	762	15–49	Antenatal care and skill delivery care
Wang 2021	(73)	Democratic Republic of the Congo	Low income	8,560	15–49	Antenatal care and skill delivery care
Dimbuene 2018	(74)	Democratic Republic of the Congo, Egypt, Ghana, Kenya, Nigeria and Zimbabwe	Low income and lower-middle income		15–49	Antenatal care and skill delivery care

*Economic classification of countries according to World Bank Classification 2022.

The number of women investigated in the included studies ranged from 200 (45) to 699,686 (61). Funnel plots were used to assess reporting bias and investigate small-study effects. The findings from funnel plots indicated symmetrical funnel plots which in turn shows the absences of publication bias (Supplementary material).

## Results of the meta-analyses

### Antenatal care utilization

#### Utilization of antenatal care by place of residence

**Figure 2:**
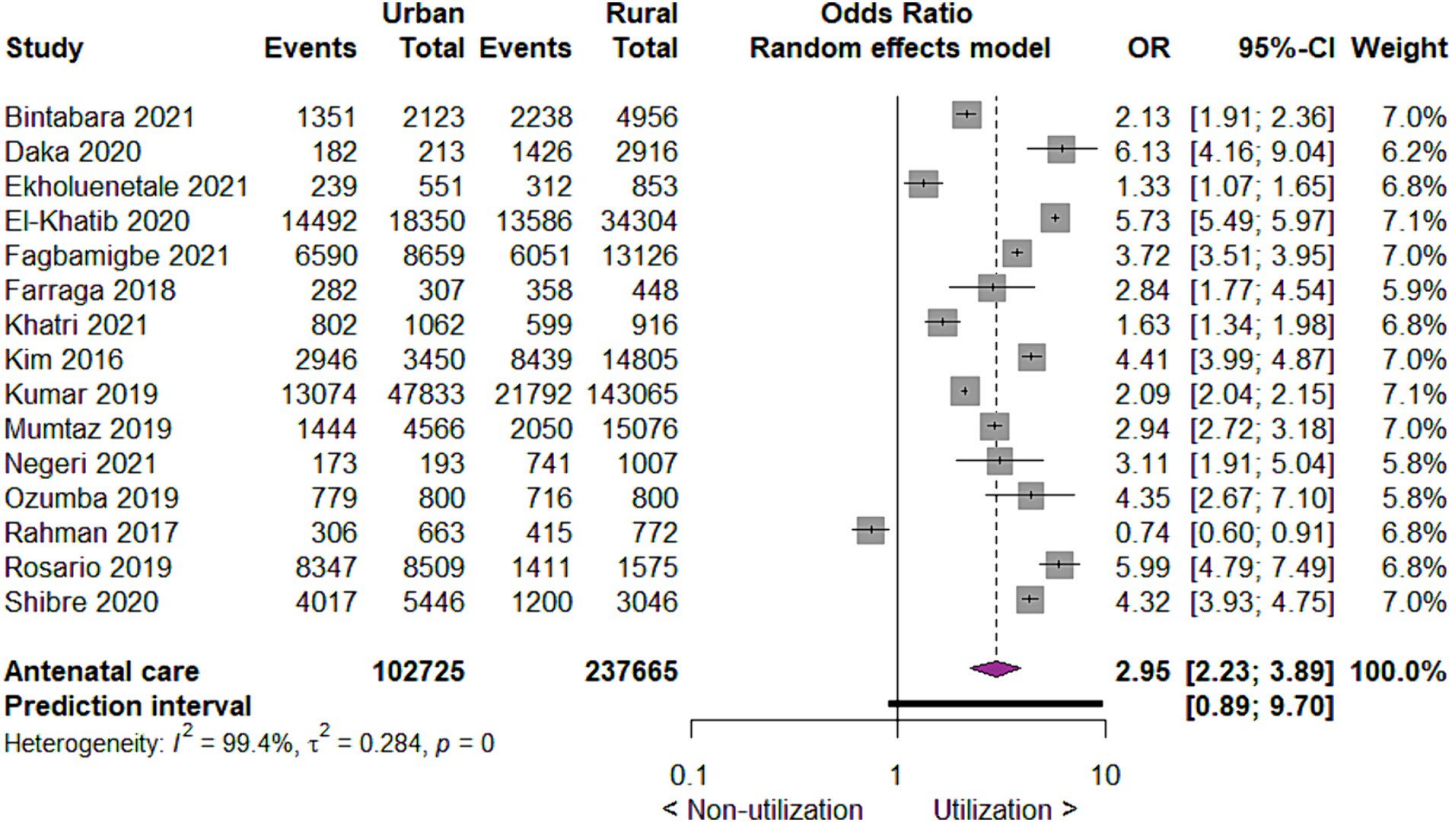
Forest plot showing antenatal care utilization by place of residence in developing countries, 2015 to 2022.

For the place of residence, utilization of antenatal care favors mothers living in urban places than those living in rural places. So, when compared to mothers living in rural areas, mothers living in urban areas utilize antenatal care three times more likely (OR 2.95; 95% CI 2.23, to 3.89; 15 studies; 340,390 participants; *p* < 0.00001) (Figure 2).

#### Utilization of antenatal care by the educational status of mothers

**Figure 3:**
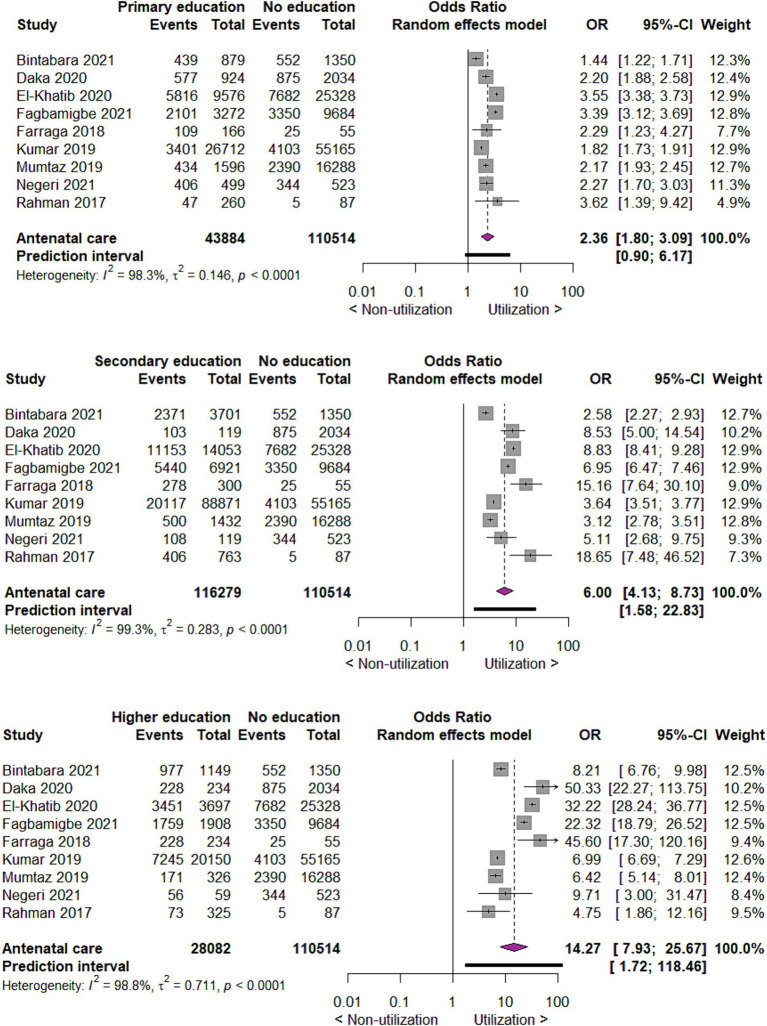
Forest plot showing antenatal care utilization by educational status of mothers in developing countries, 2015 to 2022.

For the utilization of antenatal care in relation to the educational status of the mothers, we found that compared to no education, those with primary education utilize antenatal care two times more likely (OR 2.36; 95% CI 1.80 to 3.09; 9 studies; 154,398 participants *p* < 0.00001). Similarly, compared to mothers with no education, those with secondary and higher education utilized antenatal care six times (OR 6.00; 95% CI 4.13 to 8.73; 9 studies, 154,398 participants, *p* < 0.00001) and fourteen times (OR 14.27; 95% CI 7.93, 25.67; 9 studies, 154,398 participants, *p* < 0.00001) more likely, respectively, *p* < 0.00001 (Figure 3).

#### Utilization of antenatal care by wealth index

**Figure 4:**
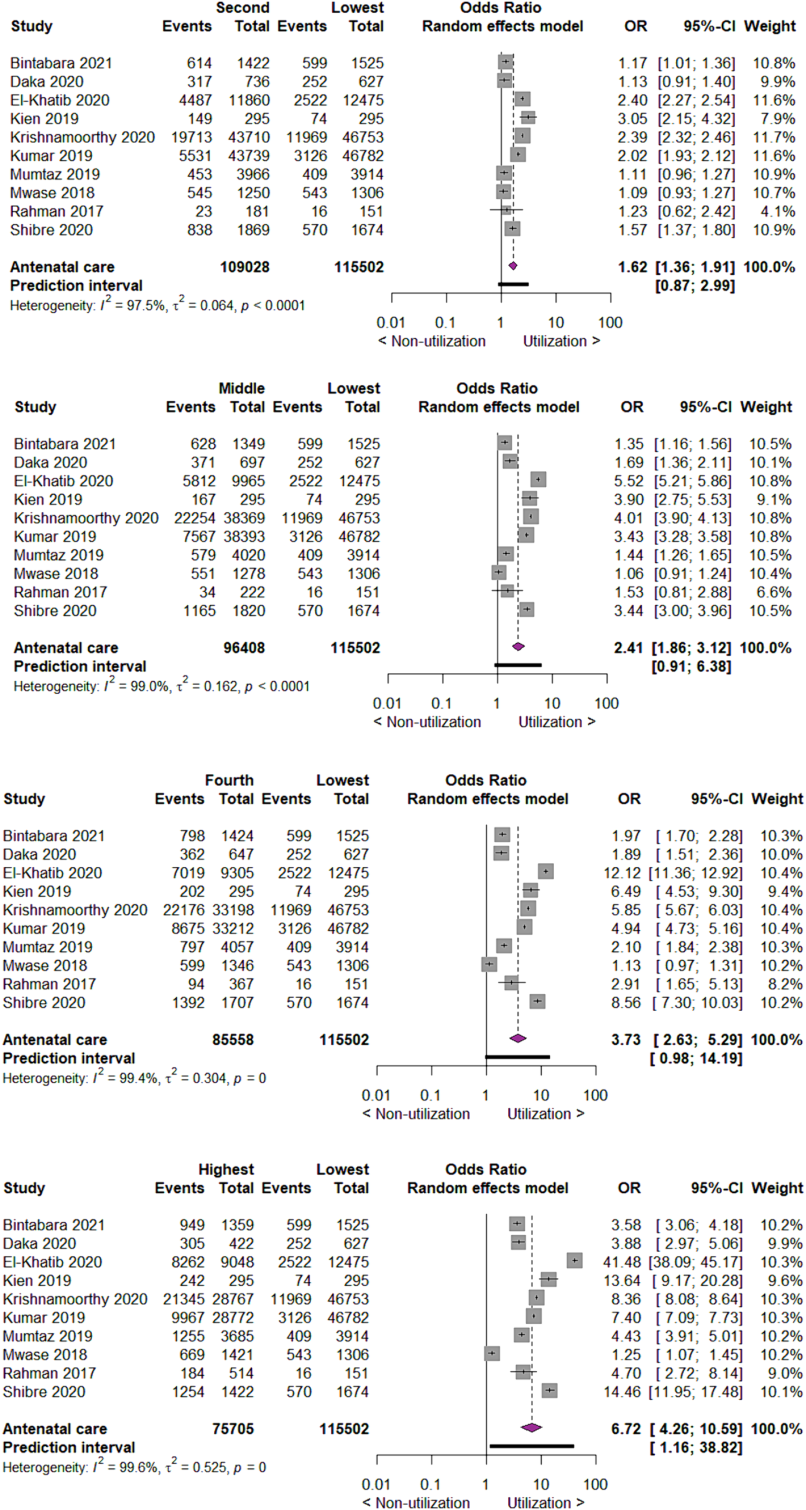
Forest plot showing antenatal care utilization by wealth index of mothers in developing countries, 2015 to 2022.

When we investigated the utilization of antenatal care by wealth index of mothers, we found that compared to mothers with the lowest wealth index, those with the second wealth index utilize antenatal care two times more likely (OR 1.62; 95% CI 1.36 to 1.91; 10 studies; 224,530 participants; *p* < 0.00001). Those in middle, fourth and highest wealth index utilize antenatal care two times (OR 2.41; 95% CI 1.86 to 3.12; 10 studies 211,910 participants; *p* < 0.00001), four times (OR 3.73; 95% CI 2.63, 5.29; 10 studies 201,060 participants; *p* < 0.00001) and seven times (OR 6.72; 95% CI 4.26, 10.59; 10 studies 191,207 participants; *p* < 0.00001) more likely as compared to mothers in lowest wealth index, respectively (Figure 4).

### Skilled delivery care utilization

#### Utilization of skilled delivery care by place of residence

**Figure 5:**
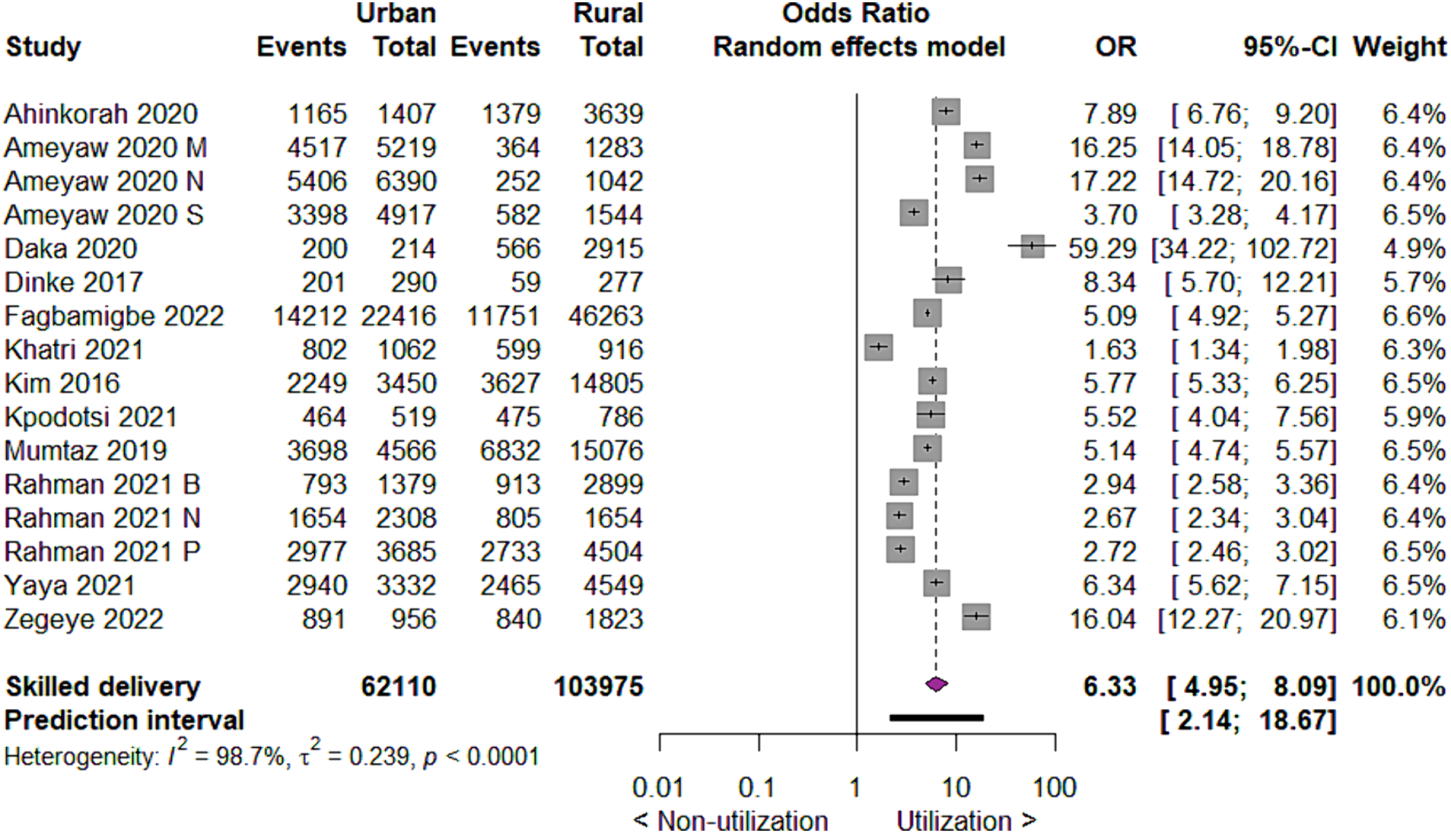
Forest plot showing skilled delivery care utilization by place of residence in developing countries, 2015 to 2022.

When we investigated the uptake of skilled delivery care by place of residence, the results indicated that mothers living in urban areas utilize skilled delivery care six times more likely than those mothers living in rural areas (OR 6.33; 95% CI 4.95 to 8.09; 16 studies, 166,085 participants; *p* < 0.00001) (Figure 5).

#### Utilization of skilled delivery care by the educational status of mothers

**Figure 6:**
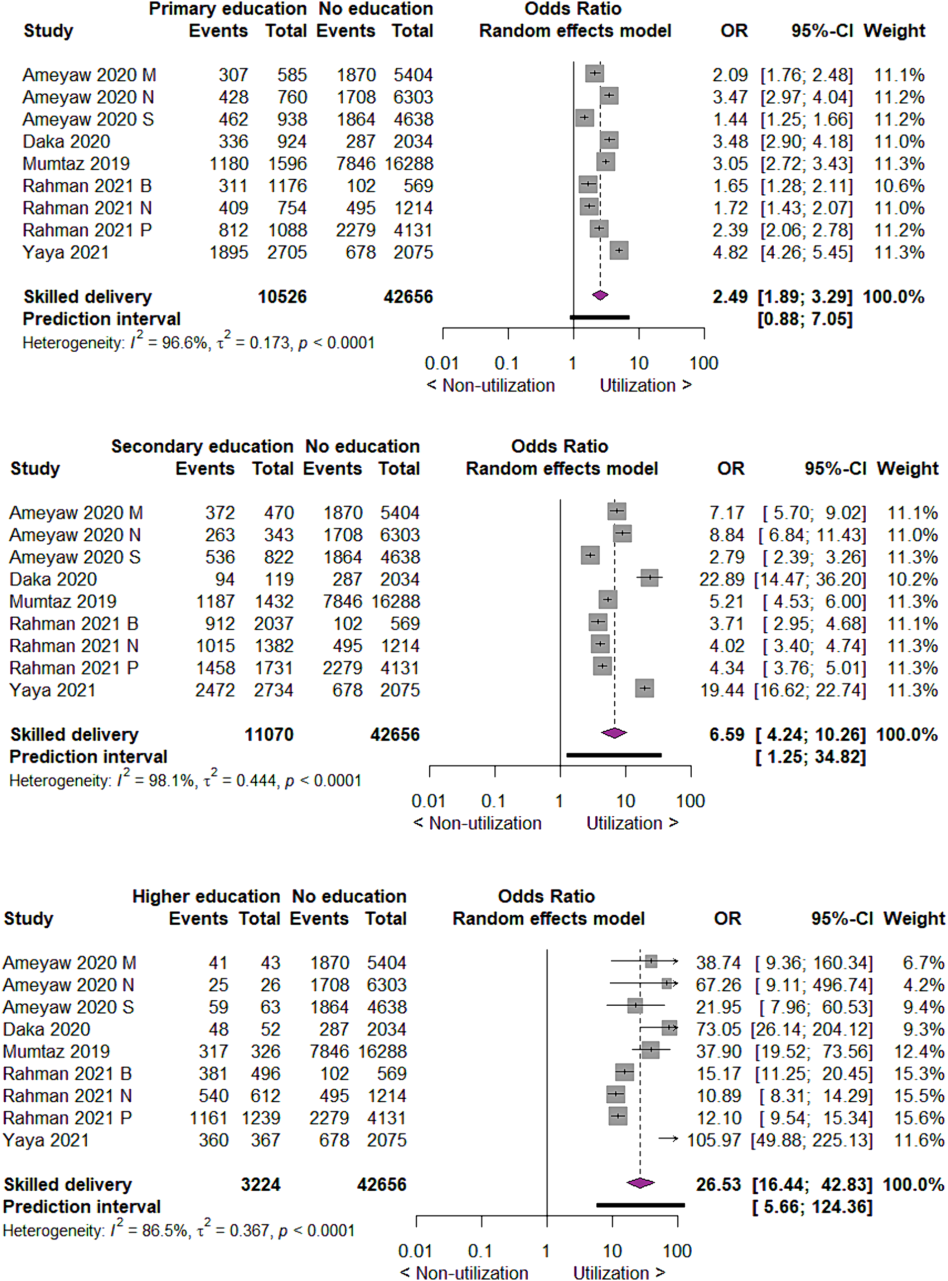
Forest plot showing skilled delivery care utilization by educational status of mothers in developing countries, 2015 to 2022.

As to the utilization of skilled delivery care by the educational status of mothers, we found that compared to those with no education, those with primary education utilized skilled delivery care three times more likely (OR 2.49; 95% CI 1.89 to 3.29; 9 studies; 53,182 participants, *p* < 0.00001). Those with secondary and higher education utilized skilled delivery care seven times (OR 6.59; 95% CI 4.24 to 10.26; 9 studies, 53,726 participants, *p* < 0.00001) and twenty-seven times (OR = 26.53; 95% CI = 16.44, 42.83; 9 studies; 45,880 participants, *p* < 0.00001) more likely than mothers with no education, respectively (Figure 6).

#### Utilization of skilled delivery care by wealth index

**Figure 7:**
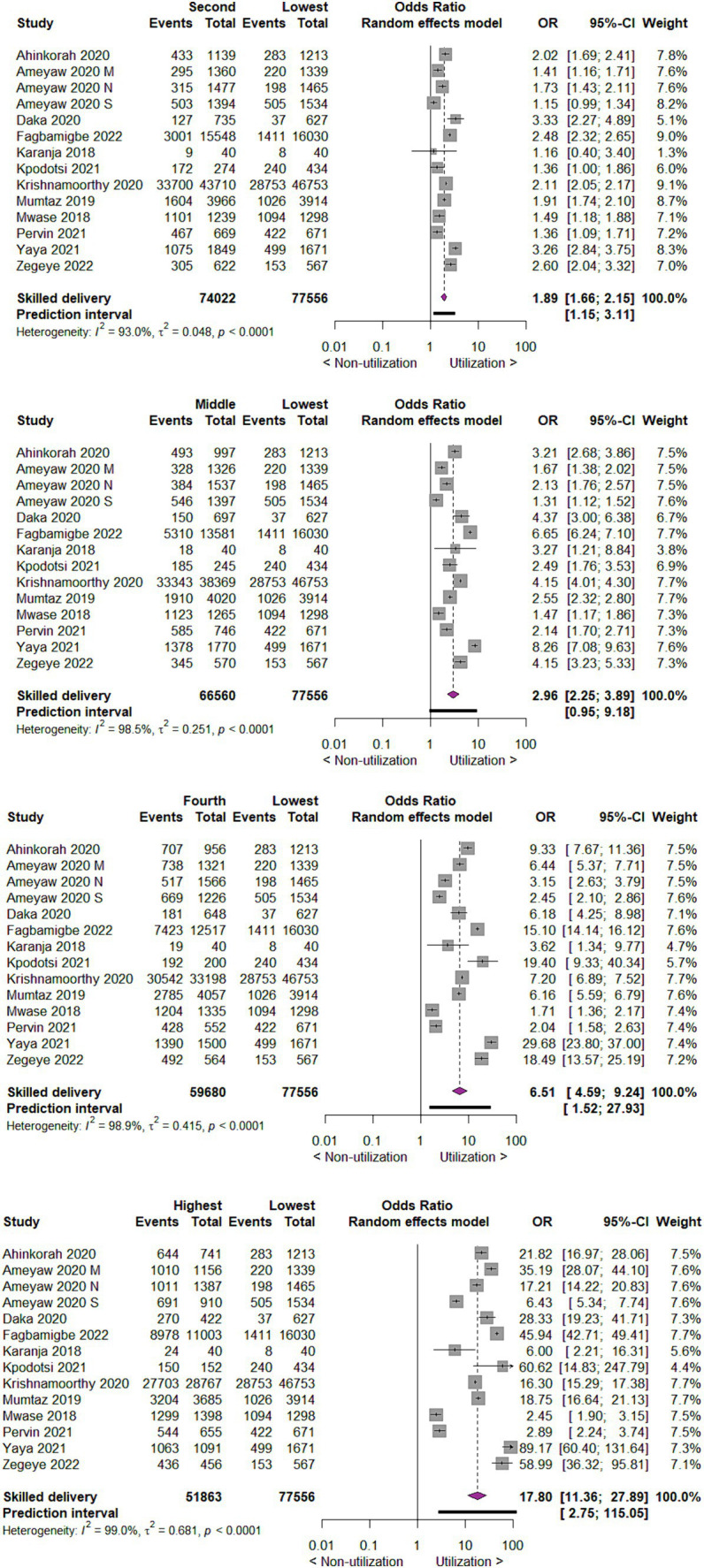
Forest plot showing skilled delivery care utilization by wealth index of mothers in developing countries, 2015 to 2022.

Concerning utilization of skilled delivery care by wealth index of mothers, we found that compared to mothers with the lowest wealth index, those with the second wealth index utilize skilled delivery care two times more likely (OR 1.89; 95% CI 1.66 to 2.15; 14 studies; 151,578 participants; *p* < 0.00001). Those mothers in the middle, fourth, and highest wealth index utilized skilled delivery care three times.

(OR 2.96; 95% CI 2.25 to 3.89; 14 studies; 144,116 participants; *p* < 0.00001), seven times (OR 6.51; 95% CI 4.59 to 9.24; 14 studies; 137,236; *p* < 0.00001) and eighteen times (OR 17.80; 95% CI 11.36 to 27.89; 14 studies; 129,419; *p* < 0.00001) are more likely as compared to mothers in lowest wealth index, respectively (Figure 7).

### Postnatal care utilization

#### Utilization of postnatal care by place of residence

**Figure 8:**
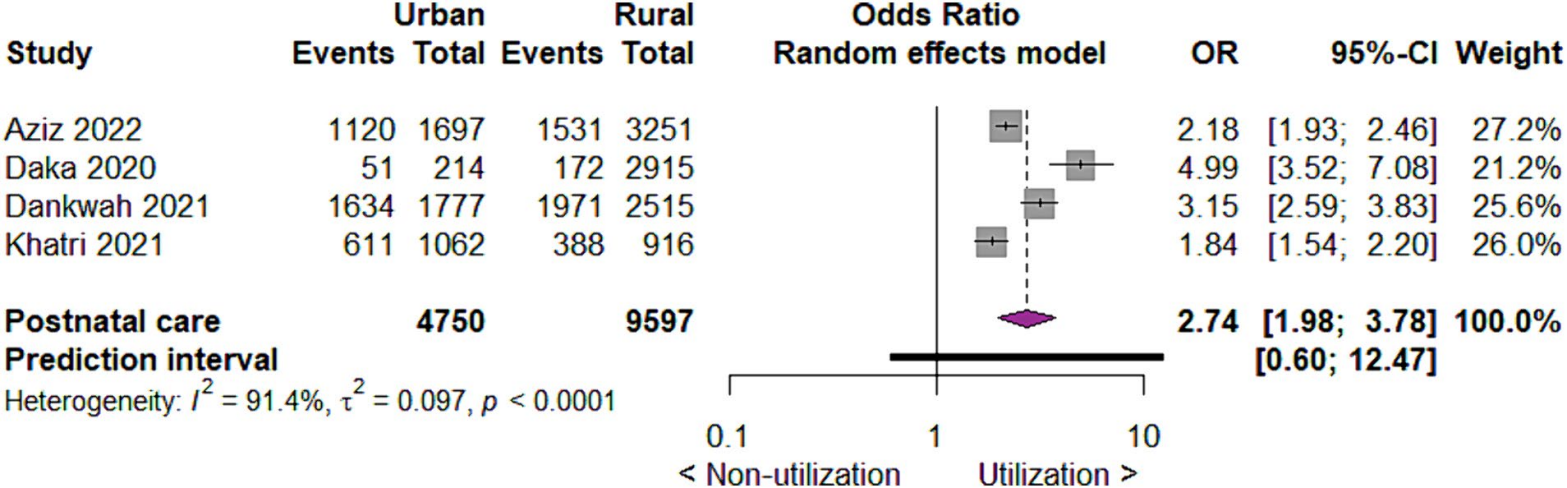
Forest plot showing postnatal care utilization by place of residence in developing countries, 2015 to 2022.

As to utilization of postnatal care by place of residence, we found that urban mothers are three times more likely to utilize postnatal care than rural mothers (OR 2.74; 95% CI 1.98 to 3.78; 4 studies; 14,347; *p* < 0.00001) (Figure 8).

#### Utilization of postnatal care by educational status of mothers

**Figure 9:**
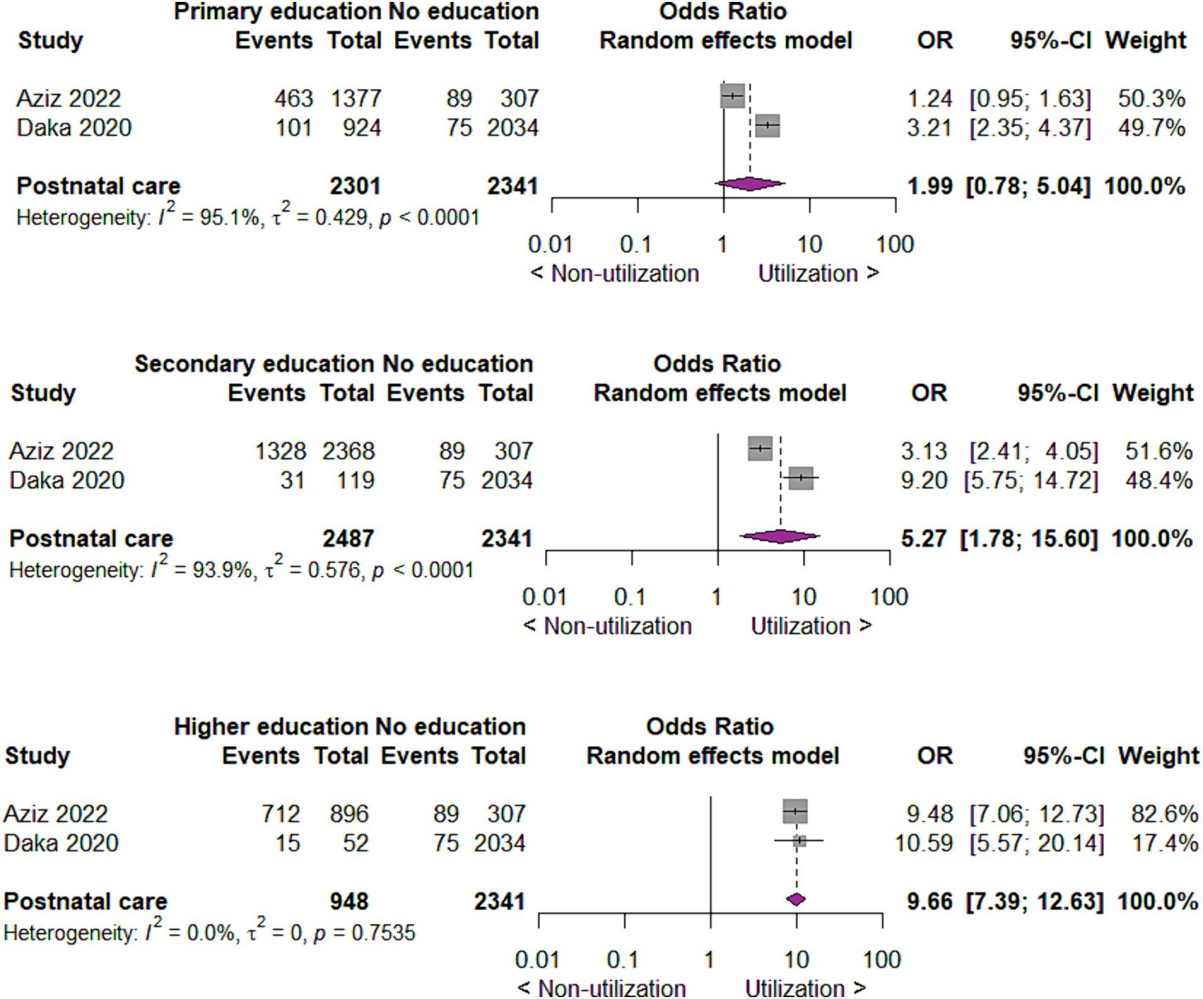
Forest plot showing postnatal care utilization by educational status of mothers in developing countries, 2015 to 2022.

Concerning the utilization of postnatal care by the educational status of mothers, we found that compared to no education, those with primary education utilize postnatal care two times more likely (OR 1.99; 95% CI 0.78 to 5.04; 2 studies; 4,642 participants; *p* = 0.15). Those with secondary education utilize postnatal care five times (OR 5.27; 95% CI 1.78 to 15.60; 2 studies; 4,828 participants; *p* = 0.003) and those with higher education ten times (OR 9.66; 95% CI 7.39 to 12.63, 2 studies; 3,289 participants; *p* < 0.00001) more likely than mothers with no education (Figure 9).

#### Utilization of postnatal care by wealth index

**Figure 10:**
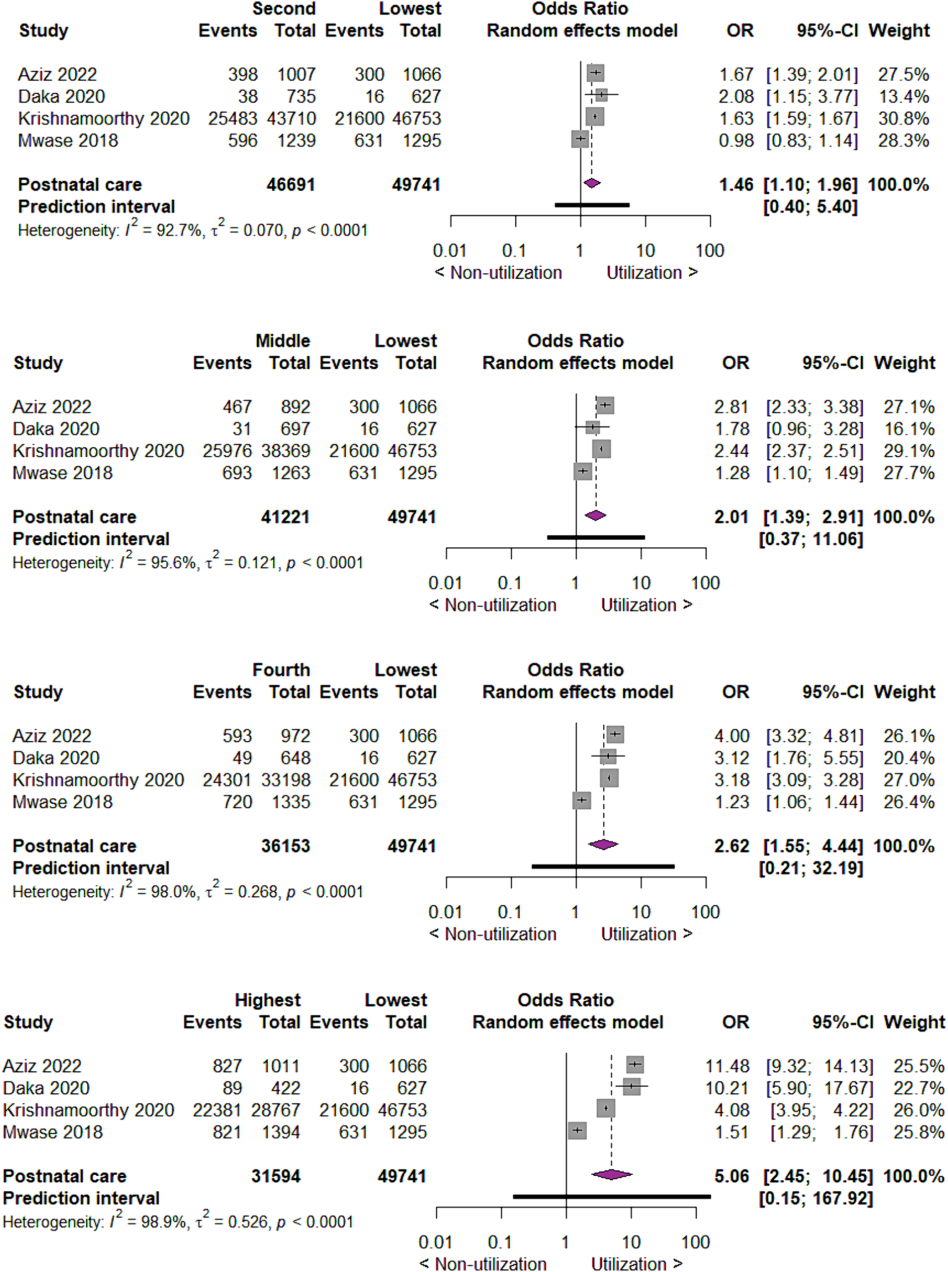
Forest plot showing postnatal care utilization by wealth index of mothers in developing countries, 2015 to 2022.

For the utilization of postnatal care by wealth index of mothers, we found that compared to mothers with the lowest wealth index, those with second wealth index utilize postnatal care two times more likely (OR 1.46; 95% CI 1.10, 1.96; 4 studies; 96,432 participants; *p* = 0.01). In the same way, compared to mothers in lowest wealth index, those in middle, fourth and highest wealth index utilize postnatal care two times (OR 2.01; 95% CI 1.39, 2.91; 4 studies; 90,962 participants; *p* = 0.0002), three times (OR 2.62; 95% CI 1.55, 4.44; 4 studies; 85,894 participants; *p* = 0.0003) and five times (OR 5.06; 95% CI 2.45, 10.45; 4 studies; 81,335 participants; *p* < 0.0001) more likely, respectively (Figure 10).

### Additional results from studies not included in meta-analyses

A total of 17 studies were not included in the meta-analysis, but their findings were summarized for the qualitative synthesis only. In these studies, the outcomes were not reported as frequencies or percentages. However, the result agrees with the result from a meta-analysis.

#### Utilization of antenatal care and skilled delivery care by the educational status of mothers

Among the 17 studies examined, 6 studies have indicated that education has a direct effect on antenatal care and skilled delivery care utilization. Notably, in Ghana, women with secondary school education or higher were more inclined to receive prenatal and skilled delivery care compared to those without formal education (59). Similarly, in Northern Ghana, the educational attainment of mothers significantly correlated with the utilization of skilled delivery services (62).

In Indonesia, evidence indicates that education plays a crucial role in influencing women to undergo more than four antenatal care visits (67). Moreover, in Congo, a higher level of education was associated with increased utilization of antenatal care (73).

A comprehensive study across five African countries demonstrated a positive link between women’s education and various aspects of maternal health care, including the type of antenatal care provider, timing and frequency of antenatal care visits, delivery location, and presence of a skilled birth attendant (74). In Nigeria, the research suggested a consistently higher concentration of maternal healthcare usage among well-educated and wealthier mothers throughout the study period (71).

#### Utilization of antenatal care and skilled delivery care by wealth index

Out of 17 studies, 10 studies have highlighted the correlation between the wealth index and the utilization of antenatal care and skilled delivery care. Research conducted in Nepal revealed an escalating trend in the utilization of skilled delivery care and antenatal care as the wealth index of mothers transitions from the poorest to the least poor (60). The ratio of utilization for four ANC visits and institutional delivery between the richest and the poorest quintile mothers were 5.08 and 9.00, respectively, (64). Additionally, women from low socioeconomic backgrounds were six times more likely to deliver without skilled assistance compared to those from high socioeconomic backgrounds (68).

In India, the probability of having at least four ANC visits is nearly four times higher for women in the richest households than for those in the poorest households (61). In the Philippines, facility-based delivery coverage was higher for non-poor households compared to poor households (63). A study across three different Asian countries revealed a pro-rich inequality in the use of facility delivery services (65).

For skilled birth attendance and four or more antenatal care visits in Kenya, the absolute difference in coverage between the wealthiest quintile (quintile 5) and poorest quintile (quintile 1) was 61.6 and 31.0%, respectively (66).

In Zimbabwe, women from middle-income and richest households were more likely to utilize antenatal care services than women from the poorest households. Maternal service utilization in Zimbabwe exhibited a pro-rich pattern, indicating a preference for maternal health care utilization among women from wealthy households (58). In Nigeria, the concentration of at least four ANC visits and a higher number of ANC visits were disproportionately higher among the rich (70). Similarly, in Myanmar, the utilization of skilled birth attendants (SBAs) among women was disproportionately concentrated among the affluent, irrespective of maternal characteristics (72).

#### Utilization of postnatal care by residence

Out of 17 studies, 1 study has highlighted the correlation between the place of residence and the utilization of postnatal care. In Sri Lanka, mothers residing in rural areas are less inclined to receive Full Postnatal care (FPNC) in comparison to their counterparts in urban areas (69).

## Discussion

In the present systematic review, we summarized up-to-date evidence about the utilization of maternal health care services, including antenatal care, skilled delivery care, and postnatal care in developing countries. In all three areas of maternal health care services, we have seen that place of residence, education, and wealth index are still important determinants, that is those living in urban areas, having a higher education level, or a higher wealth index utilize maternal health services more often. The greater the difference in education or wealth between the two groups, the greater the difference in the use of maternal health care services between them. The results of this systematic review and meta-analysis clearly indicate that there is a relative inequity in the utilization of antenatal care, skilled delivery care, and postnatal care for mothers in developing countries.

To our knowledge, this is the first systematic review summarizing available evidence on inequity in the uptake of maternal health care services in developing countries not only in a narrative way but also quantitatively, by including meta-analyses. As compared to previous systematic reviews the present systematic review does not only focus on a specific maternal health care service but includes antenatal care, skilled delivery care, as well as postnatal care. Other strengths include a comprehensive search strategy, conducted in electronic databases without imposing language restrictions. This way we think, the likelihood of overlooking published studies was minimal, although there was a potential for missing unpublished ones. We aimed to reduce bias wherever possible by having at least two review authors work independently on study selection, data extraction, and risk of bias assessments.

A previous systematic review carried out on equity in maternal healthcare service utilization in developing countries investigated studies published between 2005 and 2015 (20). The present systematic review summarizes evidence for the period 2015 to 2022. Although the results of these two systematic reviews are only partially comparable due to the more limited questioning and lack of quantitative analysis in the previous review, it can be concluded that inequity in the uptake of maternal health care services described earlier persists in developing countries.

Several interventions might be potentially effective in reducing inequalities in maternal and child health in low- and middle-income settings. Besides immunization campaigns, nutrition supplement programs, and demand-side interventions, healthcare provision improvement interventions are tested in different countries, including different health programs, which aim to improve the uptake of maternal and child healthcare services (75). In Tanzania, simple guidelines, and messages for use in primary health facilities and communities were developed (76). In Brazil, the family health program was introduced, where multi-professional teams were working under the principles of comprehensive care and provided permanent and systematic follow-up of high-risk families residing in a pre-specified area with high infant mortality rates (77). In Bangladesh, voluntary community health workers provide intensive home-based maternal and newborn care. This program aimed to increase the utilization of antenatal care and to train attendants for home delivery (78). Another program addressing both the demand side (education about the benefits of ANC and PNC visits, pathologic signs related to pregnancy and delivery) and supply side interventions (strengthening health facilities, providing trained personnel and equipment) was able to successfully improve the utilization of maternal health services and reduce inequalities (79). In Indonesia, midwives were trained and posted in every village with specific responsibility for pregnancy, delivery, and postpartum care. This way this program focused on inequities in antenatal care check-ups and birth attendance by trained professionals (80). Although the evidence level is generally low, all these programs seemed to be effective in achieving results in the field of equity of maternal and child health care services.

When designing and implementing interventions to reduce inequalities in access to maternal and newborn care, the 5As framework of access developed by Penchansky and Thomas should be borne in mind. They argue that access to care is simultaneously dependent on affordability, availability, accessibility, accommodation, and acceptability (81). These five aspects of access form a chain that is no stronger than its weakest link, so interventions that target only one or two of them may not reduce inequality at all (82, 83). In general, Changes in social determinants of health at the societal level can only be brought about by systemic, complex interventions. The obstacles to decisions leading to such measures are a lack of recognition of the problems, short-term political interests, and limited resources (84, 85).

Further research should be conducted to delve deeper into the effectiveness of the above-mentioned and additional interventions to improve the uptake of maternal health care services. The effectiveness of these interventions should be investigated separately for the different sociodemographic groups. These studies and sub-group analyses might facilitate targeted interventions in the most vulnerable groups. Governments should use the results of research activities to plan the implementation of programs. Progress in equity should be monitored, and recommendations should be forwarded to concerned bodies periodically. In most developing countries, there are demographic and health surveillance sites where demographic and health data are collected, analyzed, and reported regularly. Therefore, monitoring health inequity should be incorporated in these sites as one theme. In summary, this systematic review and meta-analysis synthesized data from 51 cross-sectional studies analyzing the uptake of maternal health care services in developing countries. The findings revealed a consistent and statistically significant effect of place of residence, education, and wealth index of mothers on the uptake of maternal health care services indicating persistence of relative inequity in utilization in developing countries. Currently, the services are mostly utilized by mothers who reside in urban areas, are more educated, and have the highest wealth index. Based on the conclusions of the literature synthesis, recommendations can be formulated for health policy that could increase the uptake of maternity services and reduce inequalities. The article may draw the attention of health policy makers to the fact that the low uptake and inequalities in maternal health care cannot be improved by interventions in the health sector alone, i.e., intersectoral action is needed.

## Data availability statement

The raw data supporting the conclusions of this article will be made available by the authors, without undue reservation.

## Author contributions

AG: Conceptualization, Data curation, Formal analysis, Investigation, Methodology, Project administration, Resources, Software, Visualization, Writing – original draft, Writing – review & editing. EM: Investigation, Methodology, Resources, Visualization, Writing – review & editing. JV: Resources, Supervision, Visualization, Writing – review & editing. SL: Formal analysis, Investigation, Methodology, Resources, Software, Supervision, Validation, Visualization, Writing – review & editing.
