# Supplementary material for: Inequity in uptake of maternal health care services in developing countries: a systematic review and meta-analysis
Source: Front Public Health. 2024 Jun 26;12:1415092. doi: 10.3389/fpubh.2024.1415092 (PMC11233804; doi:10.3389/fpubh.2024.1415092)
Supplement: Supplementary file 1 [file Table_1.DOCX]

## **SEARCH STRATEGIES**

**MEDLINE (OVID)**

1 disparit*.mp. (110895)

2 inequalit*.mp. (44355)

3 inequit*.mp. (16184)

4 equity.mp. (25337)

5 Health Status Disparities/ (18978)

6 Health Equity/ (2834)

7 exp Maternal Health Services/ (55739)

8 ((maternal or birth or childbirth or delivery or labor or maternity or pregnan* or antenatal or postnatal or perinatal or antepart* or postpart*) adj1 (care or service* or visit* or attend* or utilize or checkup*)).mp. (81942)

9 or/7-8 (114830)

10 (afghanistan or albania or algeria or american samoa or angola or "antigua and barbuda" or antigua or barbuda or argentina or armenia or armenian or aruba or azerbaijan or bahrain or bangladesh or barbados or republic of belarus or belarus or byelarus or belorussia or byelorussian or belize or british honduras or benin or dahomey or bhutan or bolivia or "bosnia and herzegovina" or bosnia or herzegovina or botswana or bechuanaland or brazil or brasil or bulgaria or burkina faso or burkina fasso or upper volta or burundi or urundi or cabo verde or cape verde or cambodia or kampuchea or khmer republic or cameroon or cameron or cameroun or central african republic or ubangi shari or chad or chile or china or colombia or comoros or comoro islands or iles comores or mayotte or democratic republic of the congo or democratic republic congo or congo or zaire or costa rica or "cote d’ivoire" or "cote d’ ivoire" or cote divoire or cote d ivoire or ivory coast or croatia or cuba or cyprus or czech republic or czechoslovakia or djibouti or french somaliland or dominica or dominican republic or ecuador or egypt or united arab republic or el salvador or equatorial guinea or spanish guinea or eritrea or estonia or eswatini or swaziland or ethiopia or fiji or gabon or gabonese republic or gambia or "georgia (republic)" or georgian or ghana or gold coast or gibraltar or greece or grenada or guam or guatemala or guinea or guinea bissau or guyana or british guiana or haiti or hispaniola or honduras or hungary or india or indonesia or timor or iran or iraq or isle of man or jamaica or jordan or kazakhstan or kazakh or kenya or "democratic people’s republic of korea" or republic of korea or north korea or south korea or korea or kosovo or kyrgyzstan or kirghizia or kirgizstan or kyrgyz republic or kirghiz or laos or lao pdr or "lao people's democratic republic" or latvia or lebanon or lebanese republic or lesotho or basutoland or liberia or libya or libyan arab jamahiriya or lithuania or macau or macao or republic of north macedonia or macedonia or madagascar or malagasy republic or malawi or nyasaland or malaysia or malay federation or malaya federation or maldives or indian ocean islands or indian ocean or mali or malta or micronesia or federated states of micronesia or kiribati or marshall islands or nauru or northern mariana islands or palau or tuvalu or mauritania or mauritius or mexico or moldova or moldovian or mongolia or montenegro or morocco or ifni or mozambique or portuguese east africa or myanmar or burma or namibia or nepal or netherlands antilles or nicaragua or niger or nigeria or oman or muscat or pakistan or panama or papua new guinea or new guinea or paraguay or peru or philippines or philipines or phillipines or phillippines or poland or "polish people's republic" or portugal or portuguese republic or puerto rico or romania or russia or russian federation or ussr or soviet union or union of soviet socialist republics or rwanda or ruanda or samoa or pacific islands or polynesia or samoan islands or navigator island or navigator islands or "sao tome and principe" or saudi arabia or senegal or serbia or seychelles or sierra leone or slovakia or slovak republic or slovenia or melanesia or solomon island or solomon islands or norfolk island or norfolk islands or somalia or south africa or south sudan or sri lanka or ceylon or "saint kitts and nevis" or "st. kitts and nevis" or saint lucia or "st. lucia" or "saint vincent and the grenadines" or saint vincent or "st. vincent" or grenadines or sudan or suriname or surinam or dutch guiana or netherlands guiana or syria or syrian arab republic or tajikistan or tadjikistan or tadzhikistan or tadzhik or tanzania or tanganyika or thailand or siam or timor leste or east timor or togo or togolese republic or tonga or "trinidad and tobago" or trinidad or tobago or tunisia or turkey or turkmenistan or turkmen or uganda or ukraine or uruguay or uzbekistan or uzbek or vanuatu or new hebrides or venezuela or vietnam or viet nam or middle east or west bank or gaza or palestine or yemen or yugoslavia or zambia or zimbabwe or northern rhodesia or global south or africa south of the sahara or sub-saharan africa or subsaharan africa or africa, central or central africa or africa, northern or north africa or northern africa or magreb or maghrib or sahara or africa, southern or southern africa or africa, eastern or east africa or eastern africa or africa, western or west africa or western africa or west indies or indian ocean islands or caribbean or central america or latin america or "south and central america" or south america or asia, central or central asia or asia, northern or north asia or northern asia or asia, southeastern or southeastern asia or south eastern asia or southeast asia or south east asia or asia, western or western asia or europe, eastern or east europe or eastern europe or developing country or developing countries or developing nation? or developing population? or developing world or less developed countr* or less developed nation? or less developed population? or less developed world or lesser developed countr* or lesser developed nation? or lesser developed population? or lesser developed world or under developed countr* or under developed nation? or under developed population? or under developed world or underdeveloped countr* or underdeveloped nation? or underdeveloped population? or underdeveloped world or middle income countr* or middle income nation? or middle income population? or low income countr* or low income nation? or low income population? or lower income countr* or lower income nation? or lower income population? or underserved countr* or underserved nation? or underserved population? or underserved world or under served countr* or under served nation? or under served population? or under served world or deprived countr* or deprived nation? or deprived population? or deprived world or poor countr* or poor nation? or poor population? or poor world or poorer countr* or poorer nation? or poorer population? or poorer world or developing econom* or less developed econom* or lesser developed econom* or under developed econom* or underdeveloped econom* or middle income econom* or low income econom* or lower income econom* or low gdp or low gnp or low gross domestic or low gross national or lower gdp or lower gnp or lower gross domestic or lower gross national or lmic or lmics or third world or lami countr* or transitional countr* or emerging economies or emerging nation?).ti,ab,sh,kf. (2226547)

11 or/1-6 (170663)

12 9 and 10 and 11 (2423)

13 limit 12 to yr="2015 -Current" (1627)

14 limit 13 to humans (1404)

**Search date: 09.06.2022**

**Results: 1404**

**Scopus (**[**www.scopus.com**](http://www.scopus.com)**)**

TITLE-ABS-KEY ( disparit*  OR  inequalit*  OR  inequit*  OR  equity )  AND  ( TITLE-ABS-KEY ( maternal  OR  birth  OR  childbirth  OR  delivery  OR  labor  OR  maternity  OR  pregnan*  OR  antenatal  OR  postnatal  OR  perinatal  OR  antepart*  OR  postpart* )  AND  TITLE-ABS-KEY ( care  OR  service*  OR  visit*  OR  attend*  OR  utilize  OR  checkup* ) )  AND  TITLE-ABS-KEY ( afghan  OR  afghans  OR  afghani  OR  albanian  OR  albanians  OR  algerian  OR  algerians  OR  "american samoan"  OR  "american samoans"  OR  angolan  OR  angolans  OR  antiguan  OR  antiguans  OR  barbudan  OR  berbudans  OR  argentine  OR  argentines  OR  argentinian  OR  argentinians  OR  argentinean  OR  argentineans  OR  armenian  OR  armenians  OR  aruban  OR  arubans  OR  azerbaijani  OR  azerbaijanis  OR  bahraini  OR  bahrainis  OR  bangladeshi  OR  bangladeshis  OR  bangalees  OR  bajan  OR  bajans  OR  belarusian  OR  belarusians  OR  byelorussian  OR  byelorussians  OR  belizean  OR  belizeans  OR  beninese  OR  benineses  OR  bhutanese  OR  bolivian  OR  bolivians  OR  bosnian  OR  bosnians  OR  botswana  OR  batswana  OR  brazilian  OR  brazilians  OR  brasilian  OR  brasilians  OR  bulgarian  OR  bulgarians  OR  burkinabe  OR  burkinese  OR  burundian  OR  burundians  OR  "cape verdean"  OR  "cape verdeans"  OR  "cabo verdean"  OR  "cabo verdeans"  OR  cambodian  OR  cambodians  OR  khmer  OR  cameroonian  OR  cameroonians  OR  "central african"  OR  "central africans"  OR  chadian  OR  chadians  OR  chilean  OR  chileans  OR  chinese  OR  colombian  OR  colombians  OR  comorian  OR  comorians  OR  congolese  OR  "costa rican"  OR  "costa ricans"  OR  ivorian  OR  ivorians  OR  croatian  OR  croatians  OR  cuban  OR  cubans  OR  cypriot  OR  cypriots  OR  czech  OR  czechs  OR  djiboutian  OR  djiboutians  OR  dominican  OR  dominicans  OR  ecuadorian  OR  ecuadorians  OR  egyptian  OR  egyptians  OR  salvadoran  OR  salvadorans  OR  "equatorial guinean"  OR  "equatorial guineans"  OR  equatoguinean  OR  equatoguineans  OR  eritrean  OR  eritreans  OR  estonian  OR  estonians  OR  swazi  OR  swazis  OR  swati  OR  swatis  OR  ethiopian  OR  ethiopians  OR  fijian  OR  fijians  OR  gabonese  OR  gabonaise  OR  gambian  OR  gambians  OR  georgian  OR  georgians  OR  ghanaian  OR  ghanaians  OR  gibraltarian  OR  gibraltarians  OR  greek  OR  greeks  OR  grenadian  OR  grenadians  OR  guamanian  OR  guamanians  OR  guatemalan  OR  guatemalans  OR  guinean  OR  guineans  OR  "bissau guinean"  OR  "bissau guineans"  OR  guyanese  OR  haitian  OR  haitians  OR  honduran  OR  hondurans  OR  hungarian  OR  hungarians  OR  indian  OR  indians  OR  indonesian  OR  indonesians  OR  iranian  OR  iranians  OR  iraqian  OR  iraqians  OR  iraqi  OR  iraqis  OR  manx  OR  jamaican  OR  jamaicans  OR  jordanian  OR  jordanians  OR  kazakhstani  OR  kazakhstanis  OR  kenyan  OR  kenyans  OR  kirabati  OR  kirabatian  OR  kirabatians  OR  "north korean"  OR  "north koreans"  OR  korean  OR  koreans  OR  kosovar  OR  kosovars  OR  kosovan  OR  kosovans  OR  kyrgyzstani  OR  kyrgyzstanis  OR  kyrgyz  OR  lao  OR  laotian  OR  laotians  OR  latvian  OR  latvians  OR  lebanese  OR  lesothan  OR  lesothans  OR  lesothonian  OR  lesothonians  OR  mosotho  OR  basotho  OR  liberian  OR  liberians  OR  libyan  OR  libyans  OR  lithuanian  OR  lithuanians  OR  macanese  OR  macedonian  OR  macedonians  OR  malagasy  OR  madagascan  OR  madagascans  OR  malawian  OR  malawians  OR  malaysian  OR  malaysians  OR  maldivian  OR  maldivians  OR  malian  OR  malians  OR  maltese  OR  marshallese  OR  marshalleses  OR  mauritanian  OR  mauritanians  OR  mauritian  OR  mauritians  OR  mexican  OR  mexicans  OR  micronesian  OR  micronesians  OR  moldovan  OR  moldovans  OR  mongolian  OR  mongolians  OR  mongol  OR  montenegrin  OR  montenegrins  OR  moroccan  OR  moroccans  OR  mozambican  OR  mozambicans  OR  burmese  OR  myanma  OR  namibian  OR  namibians  OR  nauruan  OR  nauruans  OR  nepali  OR  nepalese  OR  "netherlands antillean"  OR  "netherlands antilleans"  OR  nicaraguan  OR  nicaraguans  OR  nigerien  OR  nigeriens  OR  nigerian  OR  nigerians  OR  "northern mariana islander"  OR  "northern mariana islanders"  OR  mariana  OR  marianas  OR  omani  OR  omanis  OR  pakistani  OR  pakistanis  OR  palauan  OR  palauans  OR  panamanian  OR  panamanians  OR  "papua new guinean"  OR  "papua new guineans"  OR  paraguayan  OR  paraguayans  OR  peruvian  OR  peruvians  OR  philippine  OR  philippines  OR  philipine  OR  philipines  OR  phillipine  OR  phillipines  OR  phillippine  OR  phillippines  OR  filipino  OR  filipinos  OR  filipina  OR  filipinas  OR  polish  OR  pole  OR  poles  OR  portuguese  OR  "puerto rican"  OR  "puerto ricans"  OR  romanian  OR  romanians  OR  russian  OR  russians  OR  "soviet people"  OR  "soviet population"  OR  rwandan  OR  rwandans  OR  rwandese  OR  ruandan  OR  ruandans  OR  ruandese  OR  samoan  OR  samoans  OR  "sao tomean"  OR  "sao tomeans"  OR  santomean  OR  santomeans  OR  "saudi arabian"  OR  "saudi arabians"  OR  saudi  OR  saudis  OR  senegalese  OR  serbian  OR  serbians  OR  montenegrin  OR  montenegrins  OR  seychellois  OR  seychelloise  OR  seychelloises  OR  "sierra leonean"  OR  "sierra leoneans"  OR  slovak  OR  slovaks  OR  slovene  OR  slovenes  OR  "solomon islander"  OR  "solomon islanders"  OR  somali  OR  somalis  OR  "south african"  OR  "south africans"  OR  "south sudanese"  OR  "sri lankan"  OR  "sri lankans"  OR  ceylonese  OR  kittitian  OR  kittitians  OR  nevisian  OR  nevisians  OR  "saint lucian"  OR  "saint lucians"  OR  vincentian  OR  vincentians  OR  sudanese  OR  surinamese  OR  surinameses  OR  syrian  OR  syrians  OR  tajik  OR  tajiks  OR  tajikistani  OR  tajikistanis  OR  tanzanian  OR  tanzanians  OR  tanganyikan  OR  tanganyikans  OR  thai  OR  timorese  OR  timoreses  OR  togolese  OR  tongan  OR  tongans  OR  trinidadian  OR  trinidadians  OR  tobagonian  OR  tobagonians  OR  tunisian  OR  tunisians  OR  turk  OR  turks  OR  turkish  OR  turkmen  OR  turkmens  OR  tuvaluan  OR  tuvaluans  OR  ugandan  OR  ugandans  OR  ukrainian  OR  ukrainians  AND uruguayan  OR  uruguayans  OR  uzbek  OR  uzbeks  OR  vanuatu  OR  vanuatuan  OR  vanuatuans  OR  venezuelan  OR  venezuelans  OR  vietnamese  OR  yemeni  OR  yemenis  OR  yemenite  OR  yemenites  OR  yemenese  OR  yugoslav  OR  yugoslavs  OR  yugoslavian  OR  yugoslavians  OR  zambian  OR  zambians  OR  zimbabwean  OR  zimbabweans )  AND  PUBYEAR  >  2014

**Search date: 14.06.2022**

**Results: 27**

**Global Index Medicus (WHO)**

(disparit* OR inequalit* OR inequit* OR equity) AND ((maternal OR birth OR childbirth OR delivery OR labOR OR maternity OR pregnan* OR antenatal OR postnatal OR perinatal OR antepart* OR postpart*) AND (care OR service* OR visit* OR attend* OR utilize OR checkup*)) AND (afghan OR afghans OR afghani OR albanian OR albanians OR algerian OR algerians OR "american samoan" OR "american samoans" OR angolan OR angolans OR antiguan OR antiguans OR barbudan OR berbudans OR argentine OR argentines OR argentinian OR argentinians OR argentinean OR argentineans OR armenian OR armenians OR aruban OR arubans OR azerbaijani OR azerbaijanis OR bahraini OR bahrainis OR bangladeshi OR bangladeshis OR bangalees OR bajan OR bajans OR belarusian OR belarusians OR byelorussian OR byelorussians OR belizean OR belizeans OR beninese OR benineses OR bhutanese OR bolivian OR bolivians OR bosnian OR bosnians OR botswana OR batswana OR brazilian OR brazilians OR brasilian OR brasilians OR bulgarian OR bulgarians OR burkinabe OR burkinese OR burundian OR burundians OR "cape verdean" OR "cape verdeans" OR "cabo verdean" OR "cabo verdeans" OR cambodian OR cambodians OR khmer OR cameroonian OR cameroonians OR "central african" OR "central africans" OR chadian OR chadians OR chilean OR chileans OR chinese OR colombian OR colombians OR comorian OR comorians OR congolese OR "costa rican" OR "costa ricans" OR ivorian OR ivorians OR croatian OR croatians OR cuban OR cubans OR cypriot OR cypriots OR czech OR czechs OR djiboutian OR djiboutians OR dominican OR dominicans OR ecuadorian OR ecuadorians OR egyptian OR egyptians OR salvadoran OR salvadorans OR "equatorial guinean" OR "equatorial guineans" OR equatoguinean OR equatoguineans OR eritrean OR eritreans OR estonian OR estonians OR swazi OR swazis OR swati OR swatis OR ethiopian OR ethiopians OR fijian OR fijians OR gabonese OR gabonaise OR gambian OR gambians OR georgian OR georgians OR ghanaian OR ghanaians OR gibraltarian OR gibraltarians OR greek OR greeks OR grenadian OR grenadians OR guamanian OR guamanians OR guatemalan OR guatemalans OR guinean OR guineans OR "bissau guinean" OR "bissau guineans" OR guyanese OR haitian OR haitians OR honduran OR hondurans OR hungarian OR hungarians OR indian OR indians OR indonesian OR indonesians OR iranian OR iranians OR iraqian OR iraqians OR iraqi OR iraqis OR manx OR jamaican OR jamaicans OR jordanian OR jordanians OR kazakhstani OR kazakhstanis OR kenyan OR kenyans OR kirabati OR kirabatian OR kirabatians OR "north korean" OR "north koreans" OR korean OR koreans OR kosovar OR kosovars OR kosovan OR kosovans OR kyrgyzstani OR kyrgyzstanis OR kyrgyz OR lao OR laotian OR laotians OR latvian OR latvians OR lebanese OR lesothan OR lesothans OR lesothonian OR lesothonians OR mosotho OR basotho OR liberian OR liberians OR libyan OR libyans OR lithuanian OR lithuanians OR macanese OR macedonian OR macedonians OR malagasy OR madagascan OR madagascans OR malawian OR malawians OR malaysian OR malaysians OR maldivian OR maldivians OR malian OR malians OR maltese OR marshallese OR marshalleses OR mauritanian OR mauritanians OR mauritian OR mauritians OR mexican OR mexicans OR micronesian OR micronesians OR moldovan OR moldovans OR mongolian OR mongolians OR mongol OR montenegrin OR montenegrins OR moroccan OR moroccans OR mozambican OR mozambicans OR burmese OR myanma OR namibian OR namibians OR nauruan OR nauruans OR nepali OR nepalese OR "netherlands antillean" OR "netherlands antilleans" OR nicaraguan OR nicaraguans OR nigerien OR nigeriens OR nigerian OR nigerians OR "northern mariana islander" OR "northern mariana islanders" OR mariana OR marianas OR omani OR omanis OR pakistani OR pakistanis OR palauan OR palauans OR panamanian OR panamanians OR "papua new guinean" OR "papua new guineans" OR paraguayan OR paraguayans OR peruvian OR peruvians OR philippine OR philippines OR philipine OR philipines OR phillipine OR phillipines OR phillippine OR phillippines OR filipino OR filipinos OR filipina OR filipinas OR polish OR pole OR poles OR portuguese OR "puerto rican" OR "puerto ricans" OR romanian OR romanians OR russian OR russians OR "soviet people" OR "soviet population" OR rwandan OR rwandans OR rwandese OR ruandan OR ruandans OR ruandese OR samoan OR samoans OR "sao tomean" OR "sao tomeans" OR santomean OR santomeans OR "saudi arabian" OR "saudi arabians" OR saudi OR saudis OR senegalese OR serbian OR serbians OR montenegrin OR montenegrins OR seychellois OR seychelloise OR seychelloises OR "sierra leonean" OR "sierra leoneans" OR slovak OR slovaks OR slovene OR slovenes OR "solomon islander" OR "solomon islanders" OR somali OR somalis OR "south african" OR "south africans" OR "south sudanese" OR "sri lankan" OR "sri lankans" OR ceylonese OR kittitian OR kittitians OR nevisian OR nevisians OR "saint lucian" OR "saint lucians" OR vincentian OR vincentians OR sudanese OR surinamese OR surinameses OR syrian OR syrians OR tajik OR tajiks OR tajikistani OR tajikistanis OR tanzanian OR tanzanians OR tanganyikan OR tanganyikans OR thai OR timorese OR timoreses OR togolese OR tongan OR tongans OR trinidadian OR trinidadians OR tobagonian OR tobagonians OR tunisian OR tunisians OR turk OR turks OR turkish OR turkmen OR turkmens OR tuvaluan OR tuvaluans OR ugandan OR ugandans OR ukrainian OR ukrainians uruguayan OR uruguayans OR uzbek OR uzbeks OR vanuatu OR vanuatuan OR vanuatuans OR venezuelan OR venezuelans OR vietnamese OR yemeni OR yemenis OR yemenite OR yemenites OR yemenese OR yugoslav OR yugoslavs OR yugoslavian OR yugoslavians OR zambian OR zambians OR zimbabwean OR Zimbabweans) AND (year_cluster:[2015 TO 2022])

**Search date: 2022.06.14**

**Results: 259**

**Web of Science**

**(TI=(disparit* OR inequalit* OR inequit* OR equity)) OR (AB=(disparit* OR inequalit* OR inequit* OR equity)) 414.155**

**TI=((maternal or birth or childbirth or delivery or labor or maternity or pregnan* or antenatal or postnatal or perinatal or antepart* or postpart*) NEAR/1 (care or service* or visit* or attend* or utilize or checkup*)) OR AB=((maternal or birth or childbirth or delivery or labor or maternity or pregnan* or antenatal or postnatal or perinatal or antepart* or postpart*) NEAR/1 (care or service* or visit* or attend* or utilize or checkup*)) 90.341**

**TI=(afghan OR afghans OR afghani OR albanian OR albanians OR algerian OR algerians OR "american samoan" OR "american samoans" OR angolan OR angolans OR antiguan OR antiguans OR barbudan OR berbudans OR argentine OR argentines OR argentinian OR argentinians OR argentinean OR argentineans OR armenian OR armenians OR aruban OR arubans OR azerbaijani OR azerbaijanis OR bahraini OR bahrainis OR bangladeshi OR bangladeshis OR bangalees OR bajan OR bajans OR belarusian OR belarusians OR byelorussian OR byelorussians OR belizean OR belizeans OR beninese OR benineses OR bhutanese OR bolivian OR bolivians OR bosnian OR bosnians OR botswana OR batswana OR brazilian OR brazilians OR brasilian OR brasilians OR bulgarian OR bulgarians OR burkinabe OR burkinese OR burundian OR burundians OR "cape verdean" OR "cape verdeans" OR "cabo verdean" OR "cabo verdeans" OR cambodian OR cambodians OR khmer OR cameroonian OR cameroonians OR "central african" OR "central africans" OR chadian OR chadians OR chilean OR chileans OR chinese OR colombian OR colombians OR comorian OR comorians OR congolese OR "costa rican" OR "costa ricans" OR ivorian OR ivorians OR croatian OR croatians OR cuban OR cubans OR cypriot OR cypriots OR czech OR czechs OR djiboutian OR djiboutians OR dominican OR dominicans OR ecuadorian OR ecuadorians OR egyptian OR egyptians OR salvadoran OR salvadorans OR "equatorial guinean" OR "equatorial guineans" OR equatoguinean OR equatoguineans OR eritrean OR eritreans OR estonian OR estonians OR swazi OR swazis OR swati OR swatis OR ethiopian OR ethiopians OR fijian OR fijians OR gabonese OR gabonaise OR gambian OR gambians OR georgian OR georgians OR ghanaian OR ghanaians OR gibraltarian OR gibraltarians OR greek OR greeks OR grenadian OR grenadians OR guamanian OR guamanians OR guatemalan OR guatemalans OR guinean OR guineans OR "bissau guinean" OR "bissau guineans" OR guyanese OR haitian OR haitians OR honduran OR hondurans OR hungarian OR hungarians OR indian OR indians OR indonesian OR indonesians OR iranian OR iranians OR iraqian OR iraqians OR iraqi OR iraqis OR manx OR jamaican OR jamaicans OR jordanian OR jordanians OR kazakhstani OR kazakhstanis OR kenyan OR kenyans OR kirabati OR kirabatian OR kirabatians OR "north korean" OR "north koreans" OR korean OR koreans OR kosovar OR kosovars OR kosovan OR kosovans OR kyrgyzstani OR kyrgyzstanis OR kyrgyz OR lao OR laotian OR laotians OR latvian OR latvians OR lebanese OR lesothan OR lesothans OR lesothonian OR lesothonians OR mosotho OR basotho OR liberian OR liberians OR libyan OR libyans OR lithuanian OR lithuanians OR macanese OR macedonian OR macedonians OR malagasy OR madagascan OR madagascans OR malawian OR malawians OR malaysian OR malaysians OR maldivian OR maldivians OR malian OR malians OR maltese OR marshallese OR marshalleses OR mauritanian OR mauritanians OR mauritian OR mauritians OR mexican OR mexicans OR micronesian OR micronesians OR moldovan OR moldovans OR mongolian OR mongolians OR mongol OR montenegrin OR montenegrins OR moroccan OR moroccans OR mozambican OR mozambicans OR burmese OR myanma OR namibian OR namibians OR nauruan OR nauruans OR nepali OR nepalese OR "netherlands antillean" OR "netherlands antilleans" OR nicaraguan OR nicaraguans OR nigerien OR nigeriens OR nigerian OR nigerians OR "northern mariana islander" OR "northern mariana islanders" OR mariana OR marianas OR omani OR omanis OR pakistani OR pakistanis OR palauan OR palauans OR panamanian OR panamanians OR "papua new guinean" OR "papua new guineans" OR paraguayan OR paraguayans OR peruvian OR peruvians OR philippine OR philippines OR philipine OR philipines OR phillipine OR phillipines OR phillippine OR phillippines OR filipino OR filipinos OR filipina OR filipinas OR polish OR pole OR poles OR portuguese OR "puerto rican" OR "puerto ricans" OR romanian OR romanians OR russian OR russians OR "soviet people" OR "soviet population" OR rwandan OR rwandans OR rwandese OR ruandan OR ruandans OR ruandese OR samoan OR samoans OR "sao tomean" OR "sao tomeans" OR santomean OR santomeans OR "saudi arabian" OR "saudi arabians" OR saudi OR saudis OR senegalese OR serbian OR serbians OR montenegrin OR montenegrins OR seychellois OR seychelloise OR seychelloises OR "sierra leonean" OR "sierra leoneans" OR slovak OR slovaks OR slovene OR slovenes OR "solomon islander" OR "solomon islanders" OR somali OR somalis OR "south african" OR "south africans" OR "south sudanese" OR "sri lankan" OR "sri lankans" OR ceylonese OR kittitian OR kittitians OR nevisian OR nevisians OR "saint lucian" OR "saint lucians" OR vincentian OR vincentians OR sudanese OR surinamese OR surinameses OR syrian OR syrians OR tajik OR tajiks OR tajikistani OR tajikistanis OR tanzanian OR tanzanians OR tanganyikan OR tanganyikans OR thai OR timorese OR timoreses OR togolese OR tongan OR tongans OR trinidadian OR trinidadians OR tobagonian OR tobagonians OR tunisian OR tunisians OR turk OR turks OR turkish OR turkmen OR turkmens OR tuvaluan OR tuvaluans OR ugandan OR ugandans OR ukrainian OR ukrainians uruguayan OR uruguayans OR uzbek OR uzbeks OR vanuatu OR vanuatuan OR vanuatuans OR venezuelan OR venezuelans OR vietnamese OR yemeni OR yemenis OR yemenite OR yemenites OR yemenese OR yugoslav OR yugoslavs OR yugoslavian OR yugoslavians OR zambian OR zambians OR zimbabwean OR Zimbabweans) OR AB=(afghan OR afghans OR afghani OR albanian OR albanians OR algerian OR algerians OR "american samoan" OR "american samoans" OR angolan OR angolans OR antiguan OR antiguans OR barbudan OR berbudans OR argentine OR argentines OR argentinian OR argentinians OR argentinean OR argentineans OR armenian OR armenians OR aruban OR arubans OR azerbaijani OR azerbaijanis OR bahraini OR bahrainis OR bangladeshi OR bangladeshis OR bangalees OR bajan OR bajans OR belarusian OR belarusians OR byelorussian OR byelorussians OR belizean OR belizeans OR beninese OR benineses OR bhutanese OR bolivian OR bolivians OR bosnian OR bosnians OR botswana OR batswana OR brazilian OR brazilians OR brasilian OR brasilians OR bulgarian OR bulgarians OR burkinabe OR burkinese OR burundian OR burundians OR "cape verdean" OR "cape verdeans" OR "cabo verdean" OR "cabo verdeans" OR cambodian OR cambodians OR khmer OR cameroonian OR cameroonians OR "central african" OR "central africans" OR chadian OR chadians OR chilean OR chileans OR chinese OR colombian OR colombians OR comorian OR comorians OR congolese OR "costa rican" OR "costa ricans" OR ivorian OR ivorians OR croatian OR croatians OR cuban OR cubans OR cypriot OR cypriots OR czech OR czechs OR djiboutian OR djiboutians OR dominican OR dominicans OR ecuadorian OR ecuadorians OR egyptian OR egyptians OR salvadoran OR salvadorans OR "equatorial guinean" OR "equatorial guineans" OR equatoguinean OR equatoguineans OR eritrean OR eritreans OR estonian OR estonians OR swazi OR swazis OR swati OR swatis OR ethiopian OR ethiopians OR fijian OR fijians OR gabonese OR gabonaise OR gambian OR gambians OR georgian OR georgians OR ghanaian OR ghanaians OR gibraltarian OR gibraltarians OR greek OR greeks OR grenadian OR grenadians OR guamanian OR guamanians OR guatemalan OR guatemalans OR guinean OR guineans OR "bissau guinean" OR "bissau guineans" OR guyanese OR haitian OR haitians OR honduran OR hondurans OR hungarian OR hungarians OR indian OR indians OR indonesian OR indonesians OR iranian OR iranians OR iraqian OR iraqians OR iraqi OR iraqis OR manx OR jamaican OR jamaicans OR jordanian OR jordanians OR kazakhstani OR kazakhstanis OR kenyan OR kenyans OR kirabati OR kirabatian OR kirabatians OR "north korean" OR "north koreans" OR korean OR koreans OR kosovar OR kosovars OR kosovan OR kosovans OR kyrgyzstani OR kyrgyzstanis OR kyrgyz OR lao OR laotian OR laotians OR latvian OR latvians OR lebanese OR lesothan OR lesothans OR lesothonian OR lesothonians OR mosotho OR basotho OR liberian OR liberians OR libyan OR libyans OR lithuanian OR lithuanians OR macanese OR macedonian OR macedonians OR malagasy OR madagascan OR madagascans OR malawian OR malawians OR malaysian OR malaysians OR maldivian OR maldivians OR malian OR malians OR maltese OR marshallese OR marshalleses OR mauritanian OR mauritanians OR mauritian OR mauritians OR mexican OR mexicans OR micronesian OR micronesians OR moldovan OR moldovans OR mongolian OR mongolians OR mongol OR montenegrin OR montenegrins OR moroccan OR moroccans OR mozambican OR mozambicans OR burmese OR myanma OR namibian OR namibians OR nauruan OR nauruans OR nepali OR nepalese OR "netherlands antillean" OR "netherlands antilleans" OR nicaraguan OR nicaraguans OR nigerien OR nigeriens OR nigerian OR nigerians OR "northern mariana islander" OR "northern mariana islanders" OR mariana OR marianas OR omani OR omanis OR pakistani OR pakistanis OR palauan OR palauans OR panamanian OR panamanians OR "papua new guinean" OR "papua new guineans" OR paraguayan OR paraguayans OR peruvian OR peruvians OR philippine OR philippines OR philipine OR philipines OR phillipine OR phillipines OR phillippine OR phillippines OR filipino OR filipinos OR filipina OR filipinas OR polish OR pole OR poles OR portuguese OR "puerto rican" OR "puerto ricans" OR romanian OR romanians OR russian OR russians OR "soviet people" OR "soviet population" OR rwandan OR rwandans OR rwandese OR ruandan OR ruandans OR ruandese OR samoan OR samoans OR "sao tomean" OR "sao tomeans" OR santomean OR santomeans OR "saudi arabian" OR "saudi arabians" OR saudi OR saudis OR senegalese OR serbian OR serbians OR montenegrin OR montenegrins OR seychellois OR seychelloise OR seychelloises OR "sierra leonean" OR "sierra leoneans" OR slovak OR slovaks OR slovene OR slovenes OR "solomon islander" OR "solomon islanders" OR somali OR somalis OR "south african" OR "south africans" OR "south sudanese" OR "sri lankan" OR "sri lankans" OR ceylonese OR kittitian OR kittitians OR nevisian OR nevisians OR "saint lucian" OR "saint lucians" OR vincentian OR vincentians OR sudanese OR surinamese OR surinameses OR syrian OR syrians OR tajik OR tajiks OR tajikistani OR tajikistanis OR tanzanian OR tanzanians OR tanganyikan OR tanganyikans OR thai OR timorese OR timoreses OR togolese OR tongan OR tongans OR trinidadian OR trinidadians OR tobagonian OR tobagonians OR tunisian OR tunisians OR turk OR turks OR turkish OR turkmen OR turkmens OR tuvaluan OR tuvaluans OR ugandan OR ugandans OR ukrainian OR ukrainians uruguayan OR uruguayans OR uzbek OR uzbeks OR vanuatu OR vanuatuan OR vanuatuans OR venezuelan OR venezuelans OR vietnamese OR yemeni OR yemenis OR yemenite OR yemenites OR yemenese OR yugoslav OR yugoslavs OR yugoslavian OR yugoslavians OR zambian OR zambians OR zimbabwean OR Zimbabweans) 2.360.658**

**DOP=(2015 OR 2016 OR 2017 OR 2018 OR 2019 OR 2020 OR 2021 OR 2022) 23.537.305**

**#1 AND #2 AND #3 AND #4**

**Date of search: 14. 06. 2022**

**Results: 419**
