# Supplementary material for: Inequity in uptake of maternal health care services in developing countries: a systematic review and meta-analysis
Source: Front Public Health. 2024 Jun 26;12:1415092. doi: 10.3389/fpubh.2024.1415092 (PMC11233804; doi:10.3389/fpubh.2024.1415092)
Supplement: Supplementary file 2 [file Table_2.DOCX]

| **Section and Topic** | **Item #** | **Checklist item** | **Location where item is reported** |
| --- | --- | --- | --- |
| **TITLE** | | |  |
| Title | 1 | **Inequity in uptake of maternal health care services in developing countries: a systematic review and meta-analysis** | Title page |
| **ABSTRACT** | | |  |
| Abstract | 2 | **Background**: Maternal health service uptake remains an important predictor of maternal outcomes including Maternal mortality. This systematic review and meta-analysis aimed to summarize the available evidence on the uptake of maternal health care services in developing countries and to assess the impact of place of residence, education status, and wealth index on the uptake of these services. **Methods**: We examined the databases MEDLINE, Web of Science, Global Index Medicus, and Scopus until June 14, 2022. Cross-sectional studies done between 2015 and 2022 were considered. Mothers of reproductive age and all states of health were included in the study. Independently, two authors determined the eligibility of studies, extracted data, evaluated the risk of bias, and ranked the evidence's degree of certainty. To combine the data, we performed a random-effects meta-analysis. The PROSPERO registration ID is **CRD42022304094**. **Results**: We included 51 studies. Mothers living in urban areas were three times more likely to receive antenatal care (OR 2.95; 95% CI 2.23 to 3.89; 15 studies; 340,390 participants) than rural mothers. Compared with no education, those with primary education were twice as likely to utilize antenatal care (OR 2.36; 95% CI 1.80 to 3.09; 9 studies; 154,398 participants) and those with secondary and higher education were six and fourteen times more likely to utilize antenatal care, respectively. Mothers in the second wealth index were twice as likely as mothers in the lowest wealth index to utilize antenatal care (OR 1.62; 95% CI 1.36 to 1.91; 10 studies; 224,530 participants) and antenatal care utilization increased further among mothers in the higher wealth index. We observed similar relative inequalities in skilled delivery care and postnatal care utilization based on the pace of residence, education, and wealth index. **Conclusion**: In developing countries, the problem of inequity in utilizing maternal health care services persists and needs considerable attention. | Page 1 |
| **INTRODUCTION** | | |  |
| Rationale | 3 | Health inequality refers to a measurable aspect of health, that varies across individuals or according to socially relevant groupings [1]. These differences might exist in health conditions or might be related to access to health prevention, therapy, or rehabilitation[2]. The term ‘health inequity’ also involves the moral social perception of existing health inequalities and refers to inequalities that are considered unjust by society or that stem from some kind of injustice, mainly affecting groups with less wealth, prestige, and power [3, 4].  Maternal mortality, defined as death during pregnancy or within 42 days after childbirth, is an important indicator of socioeconomic inequalities, as it is influenced by the availability of health care and obstetric care [5]. Antenatal care contacts and births attended by skilled health professionals, early routine postnatal care, and timely management and treatment of complications are important aspects of reducing preventable maternal mortality [6, 7].  As part of the Sustainable Development Goals (SDGs), the member states of the United Nations expressed their commitment to reducing maternal mortality to less than 70 maternal deaths per 100, 000 live births by 2030 [8]. Based on recent data, we are far from reaching these goals, as since 2016 maternal mortality has been reported to decrease in only a part of the world, including central and south Asia, Australia, and New Zealand, while in Sub-Saharan Africa, Oceania, east and southeast Asia, and north Africa stagnation in maternal mortality is observed [9]. In the same period, maternal mortality increased in Europe, North America, Latin America, and the Caribbean[9]. Infectious diseases, adolescent pregnancies, cesarean section, availability of health workforce, coverage of [births](https://www.sciencedirect.com/topics/medicine-and-dentistry/childbirth) by health facilities and hospitals, and inequalities in service coverage might play the most important role in maternal mortality [10]. Underutilization of maternal and child health care services can put women and their offspring at risk of dying [11].  Worldwide, governments have started to place increasing emphasis on addressing inequities in maternal and child health services and these efforts improved relative equity of the coverage of reproductive and maternal health services across countries over the last decades [12]. However, among low and middle-income countries there are still differences in the extent to which equity is reached, and inequity in coverage of health services persists in some of the countries more than in others [13]. Even across different regions of a country, significant differences might be present in the utilization of maternal health services[14]. The most important factors indicated in studies to have an important impact on the utilization of maternal health services were education, region of residence, maternal age, and distance to health facilities [15]. For example, if a woman lives in an impoverished rural area, like Sub-Saharan Africa and South Asia, where the number of available skilled health professionals is limited, the probability of not receiving sufficient healthcare is extremely high. In high- and upper-middle-income countries more than 90% of all births are attended by a trained midwife, doctor, or nurse. In contrast, in low-income and lower-middle-income countries less than half of all births are assisted by such skilled health personnel [16].  A previous systematic review carried out on equity in maternal health care service utilization in developing countries identified 36 studies published between 2005 and 2015, out of which 33 reported severe inequities in maternal health care utilization [17]. This systematic review included only studies published in English, and maternal health care services were limited to antenatal care. Data were summarized narratively but not quantitatively. Besides, new studies have been published since then, which might show a more up-to-date picture.  For the present systematic review and meta-analysis, maternal health care services considered are antenatal care, skilled delivery care, and postnatal care, while inequity for the services is assessed based on three variables including residence, wealth index, and educational status. | Page 2-3 |
| Objectives | 4 | To summarize available evidence related to health inequity in uptake of maternal health care services in developing countries in the period of 2015 to 2022 and to assess differences across groups with different places of residence, education status, and wealth index. | Page 3 |
| **METHODS** | | |  |
| Eligibility criteria | 5 | We included cross-sectional (observational) studies investigating maternal health care services (including antenatal care, skilled delivery care, and postnatal care) utilization either separately or all services together from 2015 to 2022 in developing countries. The participants of the studies were mothers of reproductive age (15-49 years) with all health statuses, residing in and having utilized maternal health care services in developing (low and middle-income) countries. A country’s development status was determined using the World Bank Classifications of countries [19]. | Page 3 |
| Information sources | 6 | For this systematic review and meta-analysis, we searched the following electronic databases until 14th June 2022 without restrictions to the language of publications: Ovid MEDLINE, Web of Science (comprising Science Citation Index and Emerging Citation Index), Global Index Medicus (comprising African Index Medicus (AIM), Index Medicus for the Eastern Mediterranean Region (IMEMR), Index Medicus for the South-east Asia Region (IMSEAR), Latin America and the Caribbean Literature on Health Science (LILACS) and Western Pacific Region Index Medicus (WPRO) and Scopus. Details of our search strategies are available in the Supplementary Material.  We tried to identify other potentially eligible studies or supplementary publications by searching the reference lists of included studies. | Page 3 |
| Search strategy | 7 | The search strategies are attached separately as supplementary material | Supplementary Material |
| Selection process | 8 | Using Covidence^TM^ software, two review authors (GAA and EM) separately screened the titles and abstracts of each retrieved record. In a subsequent step, all possibly relevant full texts were evaluated for eligibility. Any differences of opinion were settled by consensus or by consulting a third author (SL). Articles were translated into English if necessary. | Page 3 |
| Data collection process | 9 | Using Covidence^TM^ software, two review authors (GAA and EM) separately screened the titles and abstracts of each retrieved record. In a subsequent step, all possibly relevant full texts were evaluated for eligibility. Any differences of opinion were settled by consensus or by consulting a third author (SL). Articles were translated into English if necessary. | Page 3 |
| Data items | 10a | The outcome of interest for this systematic review was inequity in utilization or uptake of maternal health care services (antenatal care, skilled delivery care, and postnatal care), reported as frequency or percentage by mothers’ residence, educational status, and wealth index. | Page 4 |
|  | 10b | We retrieved information on study methods, participants, maternal health care services, outcomes, funding sources, and potential conflict of interest statements from full-text publications. One reviewer (GAA or EM) extracted the data, while a second reviewer (GAA or EM) verified its accuracy, consistency, and completeness. | Page 4 |
| Study risk of bias assessment | 11 | Each included study's risk of bias was evaluated separately by two review authors (GAA and EM), and any discrepancies were settled by consensus. The risk of bias was evaluated with the Joanna Briggs Institute (JBI) critical appraisal checklist for analytical cross-sectional studies [20]. | Page 4 |
| Effect measures | 12 | To assess inequity, we investigated the following three factors in association with maternal health care utilization: place of residence (urban and rural), educational status (no education, primary, secondary, and higher), and wealth index (lowest, second, middle, fourth and highest). We presented the results as odds ratios (ORs) with 95% confidence intervals because our data were dichotomous. | Page 4 |
| Synthesis methods | 13a | We conducted a meta­analysis for every outcome for which we judged the participants and outcomes to be similar enough to provide meaningful results. We conducted meta-analyses for the following three comparisons: ANC utilization versus non-utilization, skilled delivery care utilization versus non-utilization, and post-natal care utilization versus non-utilization. | Page 4 |
|  | 13b | As we expected differences between studies like differences in sample size, we decided to combine the data using a random­effects model. We used Mantel­ Haenszel weighting because the outcomes are dichotomous. Results of eligible studies that do not provide data in an appropriate format for meta-analysis are summarized in a narrative format. | Page 4-5 |
|  | 13c | We used funnel plots to assess reporting bias and investigate small-study effects when at least ten studies were included in a meta­analysis. | Page 4 |
|  | 13d | We did statistical analyses using RevMan (version 5.4.1). As we expected differences between studies like differences in sample size, we decided to combine the data using a random­effects model. We used Mantel­ Haenszel weighting because the outcomes are dichotomous. | Page 4 |
|  | 13e | We assessed methodological heterogeneity by examining the risk of bias and clinical heterogeneity by examining similarities and differences between studies regarding the types of participants and outcomes. | Page 4 |
|  | 13f | We considered the size and direction of the effect and used a standard χ² test with a significance level of α=0.1 and the I² statistic, which quantifies inconsistency across studies, to assess the effect of heterogeneity on the meta­analysis. | Page 4 |
| Reporting bias assessment | 14 | Results of eligible studies that do not provide data in an appropriate format for meta-analysis are summarized in a narrative format. | Page 7 |
| Certainty assessment | 15 | As we expected differences between studies like differences in sample size, we decided to combine the data using a random­effects model. | Page 4 |
| **RESULTS** | | |  |
| Study selection | 16a | Included in the manuscript | Figure 1 |
|  | 16b | Indicated in the manuscript | Figure 1 |
| Study characteristics | 17 | Included in the manuscript | Table 2 |
| Risk of bias in studies | 18 | Assessments of risk of bias for each included study is incorporated in the manuscript | Table 1 |
| Results of individual studies | 19 | All are included in the manuscript | Figure 2-10 |
| Results of syntheses | 20a | Included in the manuscript | Table 1 |
|  | 20b | All included the manuscript | Page 5-8 |
|  | 20c | All included the manuscript | Page 4 |
|  | 20d | Present results of all sensitivity analyses conducted to assess the robustness of the synthesized results. | NA |
| Reporting biases | 21 | Included in the manuscript | Page 5 |
| Certainty of evidence | 22 | Present assessments of certainty (or confidence) in the body of evidence for each outcome assessed. | NA |
| **DISCUSSION** | | |  |
| Discussion | 23a | In the present systematic review, we summarized up-to-date evidence about the utilization of maternal health care services, including antenatal care, skilled delivery care, and postnatal care in developing countries. In all three areas of maternal health care services, we have seen that place of residence, education, and wealth index are still important determinants, that is those living in urban areas, having a higher education level, or a higher wealth index utilize maternal health services more often. The greater the difference in education or wealth between the two groups, the greater the difference in the use of maternal health care services between them. The results of this systematic review and meta-analysis clearly indicate that there is a relative inequity in the utilization of antenatal care, skilled delivery care, and postnatal care for mothers in developing countries. | Page 9 |
|  | 23b | There is a potential of missing unpublished studies | Page 9 |
|  | 23c | We may miss unpublished studies | Page 9 |
|  | 23d | Further research should be conducted to delve deeper into the effectiveness of the above-mentioned and additional interventions to improve the uptake of maternal health care services. The effectiveness of these interventions should be investigated separately for the different sociodemographic groups. These studies and sub-group analyses might facilitate targeted interventions in the most vulnerable groups. Governments should use the results of research activities to plan the implementation of programs. Progress in equity should be monitored, and recommendations should be forwarded to concerned bodies periodically. In most developing countries, there are demographic and health surveillance sites where demographic and health data are collected, analyzed, and reported regularly. Therefore, monitoring health inequity should be incorporated in these sites as one theme. | Page 10 |
| **OTHER INFORMATION** | | |  |
| Registration and protocol | 24a | This study is registered with PROSPERO with registration ID: **CRD42022304094**. | Page 3 |
|  | 24b | The protocol can be accessed from PROSPERO | Page 3 |
|  | 24c | Describe and explain any amendments to information provided at registration or in the protocol. | NA |
| Support | 25 | No sources of financial support | Page 10 |
| Competing interests | 26 | No competing interest | Page 10 |
| Availability of data, code and other materials | 27 | Data can be made available on reasonable request | Page 10 |

*From:*  Page MJ, McKenzie JE, Bossuyt PM, Boutron I, Hoffmann TC, Mulrow CD, et al. The PRISMA 2020 statement: an updated guideline for reporting systematic reviews. BMJ 2021;372:n71. doi: 10.1136/bmj.n71

For more information, visit: <http://www.prisma-statement.org/>


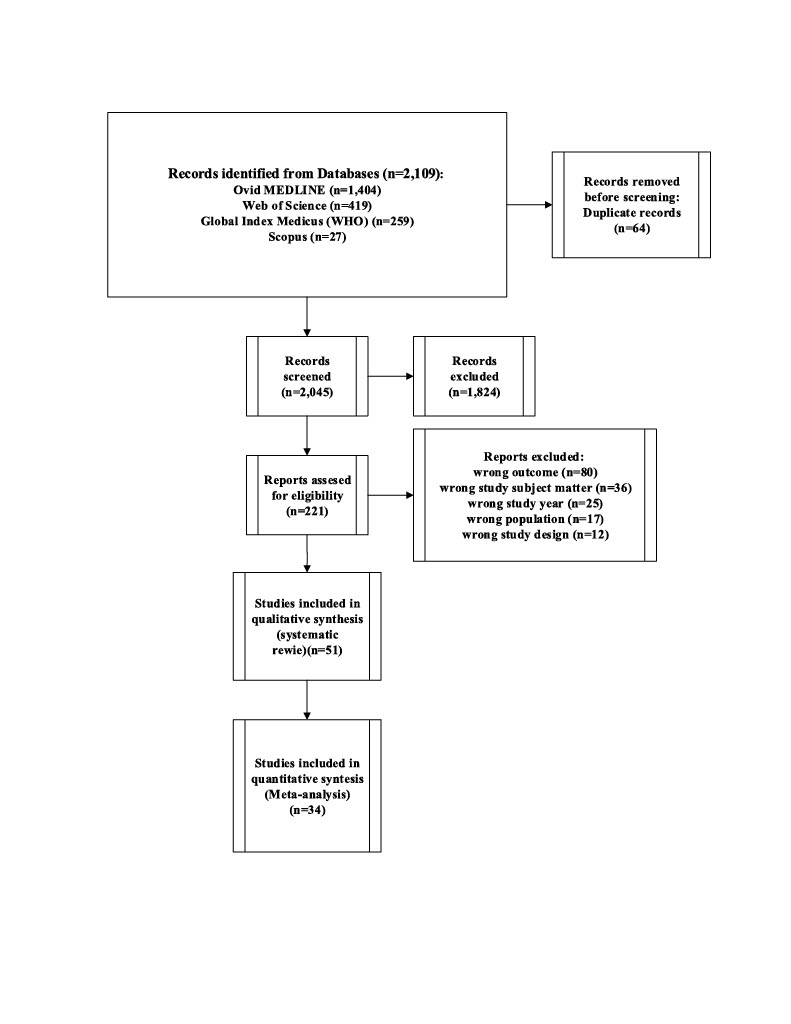


Figure 1: PRISMA flow-diagram indicating study selection.
